# Cigarette Smoke Contributes to the Progression of MASLD: From the Molecular Mechanisms to Therapy

**DOI:** 10.3390/cells14030221

**Published:** 2025-02-04

**Authors:** Jiatong Xu, Yifan Li, Zixuan Feng, Hongping Chen

**Affiliations:** 1 Queen Mary School, Medical College, Nanchang University, Nanchang 330006, China; jiatong.xu@se21.qmul.ac.uk (J.X.); yifan.li@se21.qmul.ac.uk (Y.L.); z.feng@se21.qmul.ac.uk (Z.F.); 2 Department of Histology and Embryology, Jiangxi Medical College, Nanchang University, Nanchang 330019, China

**Keywords:** cigarette smoke, MASLD, MASH, liver fibrosis, smoking cessation, lifestyle intervention, natural products

## Abstract

Cigarette smoke (CS), an intricate blend comprising over 4000 compounds, induces abnormal cellular reactions that harm multiple tissues. Non-alcoholic fatty liver disease (NAFLD) is a prevalent chronic liver disease (CLD), encompassing non-alcoholic fatty liver (NAFL), non-alcoholic steatohepatitis (NASH), cirrhosis, and hepatocellular carcinoma (HCC). Recently, the term NAFLD has been changed to metabolic dysfunction-associated steatotic liver disease (MASLD), and NASH has been renamed metabolic dysfunction-associated steatohepatitis (MASH). A multitude of experiments have confirmed the association between CS and the incidence and progression of MASLD. However, the specific signaling pathways involved need to be updated with new scientific discoveries. CS exposure can disrupt lipid metabolism, induce inflammation and apoptosis, and stimulate liver fibrosis through multiple signaling pathways that promote the progression of MASLD. Currently, there is no officially approved efficacious pharmaceutical intervention in clinical practice. Therefore, lifestyle modifications have emerged as the primary therapeutic approach for managing MASLD. Smoking cessation and the application of a series of natural ingredients have been shown to ameliorate pathological changes in the liver induced by CS, potentially serving as an effective approach to decelerating MASLD development. This article aims to elucidate the specific signaling pathways through which smoking promotes MASLD, while summarizing the reversal factors identified in recent studies, thereby offering novel insights for future research on and the treatment of MASLD.

## 1. Introduction

Non-alcoholic fatty liver disease (NAFLD) is a chronic liver disease (CLD) characterized by steatosis, cellular damage, and fibrosis [1,2,3,4]. It is a generic term for several forms of diseases: non-alcoholic fatty liver (NAFL), non-alcoholic steatohepatitis (NASH), cirrhosis, and hepatocellular carcinoma (HCC) [5,6]. Recently, the term NAFLD has been changed to metabolic dysfunction-associated steatotic liver disease (MASLD), and NASH has been renamed metabolic dysfunction-associated steatohepatitis (MASH) [7,8]. These changes emphasize the importance of metabolic dysfunction in the development of these diseases and the change in the description of these diseases, as “steatosis” rather than “fat”, reduces the stigma attached to the patient [8,9]. Despite the change in terminology, previous NAFLD-related findings still apply to MASLD [1]. In fact, the occurrence and development of MASLD are closely related to metabolic syndromes, such as dyslipidemia, diabetes mellitus, and hypertension [10]. Dyslipidemia is capable of inducing simple steatosis in the liver, which subsequently induces pathophysiological changes, such as oxidative stress, inflammatory responses, abnormal organelle function, and apoptosis, thereby promoting the progression of MASLD to MASH and cirrhosis [11,12]. Diabetes-related insulin resistance is an important source of hepatic de novo lipogenesis (DNL), because the ongoing metabolic catabolism of fat associated with insulin resistance increases the circulating levels of free fatty acids (FFAs), and these FFAs are readily translocated to the liver and lead to lipotoxicity, which provides the conditions for the development of MASLD [13,14]. Hypertension causes MASLD, even in the absence of other risk factors, and blood pressure levels are strongly associated with the severity of the disease [15]. In addition to metabolic factors, alcohol is involved in the development and progression of MASLD and is the most important predictor of mortality in MASLD [16].

MASLD is recognized as the fastest growing cause of CLD in the US and worldwide [17]. Currently, approximately 38% of the adult population and 7–14% of children and adolescents suffer from MASLD [16]. Estimates suggest that by 2040, MASLD is expected to affect over 55% of adults [16]. Due to the increasing burden of MAFLD/MASH and its severe consequences, the need for effective treatment is more urgent than ever. However, an effective officially approved medical treatment has not yet been established [18,19,20]. Despite the antioxidant function of vitamin E, which plays a role in improving MASH, it does not control the progression of liver fibrosis and, therefore, is not an ideal therapeutic option [21]. Currently, improvements in lifestyle remain the first line of treatment for patients with MASLD [22].

Several studies have confirmed that smoking is a significant risk factor for MASLD: the incidence rate is higher in smokers versus non-smokers [16,23,24]. A large Korean cohort study involving 5,080,410 participants, with 39,910,331 person-years of follow-up, found that past or current smokers were more likely to have persistent MASLD [25]. When referring to the impact of smoking on lipid metabolism, Martins-Green et al. observed that in the livers of mice exposed to cigarette smoke (CS), the level of low-density lipoprotein (LDL; a type of cholesterol that has a detrimental effect on the organism) was significantly elevated [26]. It is worth noting that smoking induces hepatocyte damage and tissue necrosis. Yang et al. demonstrated that the maternal administration of mainstream cigarette smoke (MSCS) increased the levels of serum alanine aminotransferase (ALT) and serum aspartate aminotransferase (AST), markers of liver injury [27]. Furthermore, various studies have confirmed that the progression of MASLD is often accompanied by extensive irreversible fibrosis [28,29], and smoking accelerates this process. For instance, a cross-sectional cohort study discovered an association between the number of packs of cigarettes smoked per year and the severity of liver fibrosis in patients with MASLD [30]. When MASH progresses to the end stage of the disease, the therapeutic effect is limited, thus making MASH the primary indication for liver transplantation [31,32,33]. Active smoking is associated with increased all-cause mortality and reduced healing effects in patients after liver transplantation, with estimated 1-, 5-, and 10-year survival rates of 94%, 68%, and 54%, respectively, compared with 94%, 83%, and 77% for non-smokers (*p* = 0.04) [34]. In light of the significant worsening of MASLD by smoking, the positive effects of smoking cessation are important. Multivariate logistic regression analyses, performed by Takenaka et al., demonstrated that an increase in the time taken to quit smoking was significantly and negatively associated with the prevalence of moderate-to-severe MASLD [35]. Surprisingly, it has been reported that smoking cessation may increase the treatment response, reduce fibrosis spread and HCC incidence, and improve liver transplantation outcomes [36]. Overall, smoking increases mortality in patients with MASLD [24] and the risk associated with MASLD may, therefore, be reduced by smoking cessation, which may be a promising way to reduce the incidence of MASLD through improving the prognosis of patients.

Although the correlation between smoking and MASLD progression has been continuously demonstrated, the specific signaling pathways involved have not been comprehensively summarized. In this review, we aim to explore the molecular mechanisms underlying the contribution of smoking to MASLD and to propose reliable reversal factors. Ultimately, our goal was to provide constructive suggestions for lifestyle modifications by patients, with the aim of halting the progression of MASLD.

## 2. Smoking Promotes MASLD by Disrupting Lipid Metabolism

The excessive accumulation of fatty acids in the liver is a distinguishing feature of MASLD, which ultimately leads to excess triglycerides, the most significant cause of hepatic steatosis [37]. Multiple studies have shown that smoking is a risk factor for disturbing the homeostasis of lipid metabolism in the human liver. In a recent multivariate logistic regression analysis, conducted in South Korea, the concurrent use of e-cigarettes and conventional cigarettes was found to be associated with hepatic steatosis in MASLD [38]. Hellerstein et al. demonstrated that acute smoking (two cigarettes per hour) significantly elevated human plasma FFA concentrations and increased the risk of MASLD, using stable isotope tracers [39]. A National Health Survey in Chile identified smoking as one of the risk factors for increased lipid accumulation products (LAPs) in MASLD [40]. A cross-sectional study of U.S. adults confirmed that smoking increases the ratio of triglycerides to high-density lipoprotein cholesterol (TG/HDL-C), an indicator of worsening MASLD [41]. In addition, a large health management cohort study found a correlation between an increased triglyceride–glucose (TyG) index from smoking and the risk of MASLD progression [42]. The abundant basic experiments conducted on animal models of MASLD have led to the identification of additional molecular mechanisms associated with smoking and aberrant lipid metabolism in MASLD. By sorting out and analyzing the internal logic of the various alterations, we can more thoroughly understand the effects of smoking on MASLD and, thus, find potential therapeutic targets for improving the deterioration of chronic liver disease caused by smoking.

### 2.1. Smoking Increases the Risk of MASLD by Altering the AMPK-Related Signaling Pathway

**Figure 1:**
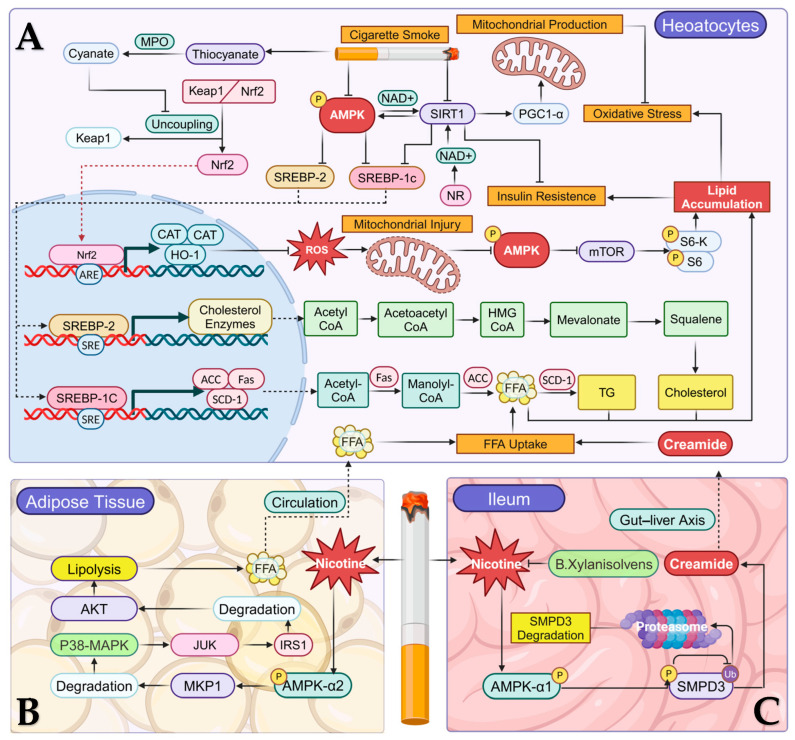
In MASLD, CS affects the AMPK signaling pathway in different ways in the liver (**A**), adipose tissue (**B**), and ileum (**C**) to perturb lipid metabolism. The solid arrows represent ‘facilitation’, the dashed arrows in different colors represent ‘transport’, and the solid ‘T’ lines represent ‘inhibition’. Abbreviations: CS: cigarette smoke; MPO: myeloperoxidase; Keap1: Kelch-like ECH-associated protein 1; Nrf2: nuclear factor erythroid 2-related factor 2, ARE: antioxidant response element; CAT: catalase; SOD: superoxide dismutase; HO-1: heme oxygenase-1; ROS: reactive oxygen species; AMPK: AMP-activated protein kinase; mTOR: mammalian target of rapamycin; pS6-K: p70 ribosomal S6 kinase; pS6: phosphorylated S6 ribosomal protein; SREBP: sterol regulatory element-binding protein; NAD+: nicotinamide adenine dinucleotide; Sirt1: Sirtuin 1; PGC1-α: peroxisome proliferator-activated receptor-γ co-activator 1; NR: nicotinamide riboside; SRE: sterol response elements; Acetyl CoA: acetyl coenzyme A, Acetoacetyl CoA: acetoacetyl coenzyme A; HMG CoA: 3-hydroxy-3-methyl glutaryl coenzyme A; ACC: acetyl CoA carboxylase; Fas: fatty acid synthases; SCD1: stearoyl-CoA desaturase-1; Malonyl CoA: malonyl coenzyme A; FFA: free fatty acid; MKP1: MAP kinase phosphatase-1; P38-MAPK: p38 mitogen-activated protein kinase; JNK: c-Jun-NH 2 -terminal kinase; IRS1: insulin receptor substrate 1; AKT: protein kinase B; SMPD-3: sphingomyelin phosphodiesterase 3; B. xylanisolvens: Bacteroides xylanisolvens. (This figure was created with biorender.com, accessed on 3 February 2025).

AMP-activated protein kinase (AMPK) is widespread in various eukaryotic cells. It is an evolutionarily highly conserved heterotrimeric serine/threonine protein kinase [43]. As a cellular energy sensor and a major regulator of hepatic lipid function, it plays an integral role in the regulation and control of MASLD [44,45]. The phosphorylation site of the α-subunit is in the activation loop of the N-terminal kinase structural domain, where the phosphorylation of threonine (Thr172) is indispensable for the full activation of AMPK [46]. Numerous studies have proved that CS can affect AMPK function, thus influencing diverse signaling pathways to promote MASLD, as depicted in Figure 1.

#### 2.1.1. AMPK/SREBP Signaling Pathway

An immunoblot analysis proved that after hepatocytes were exposed to an SSW smoke (sidestream whole smoke, the primary constituent of “second-hand” smoke) solution for 10 min, the phosphorylation level of AMPK in hepatocytes decreased and gradually returned to a normal activity level after two hours [47]. This implies that the activity of AMPK in hepatocytes is suppressed by SSW smoke. According to the report, CS-induced AMPK inhibition reduced Ser372 phosphorylation in sterol-regulatory element-binding protein-1c (SREBP1c), thereby activating it [47,48,49]. Interestingly, the activation and expression of SREBP-1c by SSW smoke were dose dependent: the higher the concentration of SSW smoke, the greater the activation of SREBP-1c in SSW smoke solutions at dilutions of 1:20, 1:40, and 1:80 [47]. Upon activation, SREBP-1c translocates into the nucleus and exerts its regulatory role by binding to sterol response elements (SREs) present within the promoter/enhancer regions of the acetyl CoA carboxylase (ACC), fatty acid synthases (Fas), and stearoyl-CoA desaturase-1 (SCD1) genes, thereby facilitating their expression [50,51]. ACC promotes the de novo synthesis of lipids in MASLD, i.e., the conversion from acetyl CoA to malonyl CoA, and Fas catalyzes the subsequent transformation of malonyl CoA to FAAs [52]. SCD-1 is a key enzyme in the conversion of FFAs to triglycerides [53]. In addition, AMPK in the liver can also directly phosphorylate and inactivate ACC [54,55], whereas the downregulation of AMPK attenuates this effect. The AMPK/SREBP-2 signaling pathway plays a crucial role in the regulation of hepatic de novo cholesterol synthesis [56]. Hepatic cholesterol overload-mediated lipotoxicity has been reported to be a mechanistic contributor to MASLD progression [57]. Exposure to MSCS can suppress AMPK activity and induce the upregulation of the SREBP-2 mRNA [27]. SREBP-2 exerts regulatory control over the expression of key enzymes, including HMG-CoA synthase, HMG-CoA reductase, farnesyl diphosphate synthase, and squalene synthase [50]. These components play pivotal catalytic roles in various aspects of the hepatic cholesterol synthesis cascade.

Notably, the study performed by Yuan et al. only confirmed that SSW smoke could affect the function of SREBP-1c rather than SREBP-2 in male apoB100 transgenic mice [47]. However, Yang et al. revealed a simultaneous upregulation of SREBP-2 and SREBP-1c mRNA expression in offspring mice following maternal exposure to mainstream smoke (MSS) [27]. This discrepancy in the findings may stem from the variations in smoke administration methods among the mice, as the toxicity and mutagenicity of SSW smoke significantly diverges from that of MSS [58,59] and the extent of the cell damage is different. In addition, the changes in AMPK/SREBP-1c during CS exposure remain controversial. Hasan et al. found that the phosphorylation of AMPK, as well as the expression of SREBP1c, FAS, and ACC in the liver of mice, did not change significantly after exposure to electronic nicotine delivery systems (ENDSs) compared to the control groups [60]. The observed outcome could potentially be attributed to inadequate exposure concentrations of the noxious constituents in smoke or the limited duration of an individual smoking session. However, for the human body, smoking is often a long-term process that is difficult to stop. The hepatic injury induced by smoking necessitates prolonged accumulation. In conclusion, CS can induce de novo lipid synthesis in the liver to promote MASLD through the AMPK/SREBP signaling pathway.

#### 2.1.2. AMPK/SIRT1 Signaling Pathway

Sirtuin 1 (SIRT1) is a nicotinamide adenine (NAD+)-dependent histone deacetylase [61], which has a positive effect on the control of MASLD [62]. It was confirmed that AMPK has the capability to increase the level of NAD+, thereby enhancing the activity of SIRT1 [63,64]. AMPK and SIRT1 are also able to interact with each other and co-regulate the homeostasis of hepatic lipid metabolism [65]. A combined Western blot and SDS immunoprecipitation study revealed that SIRT1 could inhibit de novo lipid synthesis through the direct deacetylation of SREBP-1c at Lys-289 and Lys-309 sites [66]. Moreover, the AMPK/SIRT1/PGC1-α signaling pathway promotes lipid metabolism by enhancing intracellular mitochondrial production [67,68,69]. SIRT1 also promotes the synthesis of the liver-derived hormone Fibroblast growth factor 21 (FGF21) [70]. FGF21 restores glucose and lipid homeostasis and insulin sensitivity in the body [70]. Based on these results, we can conclude that SIRT1 has a critical protective role against MASLD.

Previously, a decrease in both AMPK and SIRT1 levels was observed in the liver lysates of mice receiving nicotine injections [71]. A recent study confirmed that nicotinamide riboside (NR), a precursor of NAD+ [72], exerts a restorative effect on hepatic steatosis, oxidative stress, and mitochondrial damage induced by nicotine plus Coca-Cola, through the activation of SIRT1/PGC1-α levels [73]. This experiment may provide evidence that NR has the potential to mitigate MASLD progression caused by nicotine, by enhancing metabolism. However, both nicotine and Coca-Cola can cause hepatic steatosis, the two risk factors are combined to induce model organisms, which risk factor the NR causes a direct reversal of the consequences and needs further investigations.

#### 2.1.3. AMPK/mTOR Signaling Pathway

Recent studies have proven that CS is one of the main ways in which the body produces thiocyanate [74,75]. Smokers tend to have higher serum thiocyanate levels than non-smokers and ex-smokers [76]. Moreover, persistent smoking induces an inflammatory response in the liver [77], and the concomitant occurrence of smoking plus inflammation encourages myeloperoxidase (MPO) to catalyze the oxidation of thiocyanate, resulting in the production of cyanate at the site of inflammation [78]. These findings illustrate the contribution of smoking to organismal cyanate production through both precursor production and catalysis. Interestingly, cyanate has been reported to cause oxidative stress and hepatic lipid accumulation by affecting the AMPK/mTOR signaling pathway in the liver.

Cyanate exposure affects lipid metabolism in the liver. Nuclear factor erythroid 2-related factor 2 (NRF2) is a transcription factor that mitigates the inflammatory response, lipotoxicity, and oxidative stress prevalent in MASLD [79]. Cyanate maintains the Nrf2 transcriptional silencing state by stabilizing the complex formed by NRF2 with Kelch-like ECH-associated protein 1 (Keap1) and, as a result, Nrf2 loses its ability to enter the nucleus and bind to the antioxidant response element (ARE) [80,81]. This downregulates the expression of three antioxidant enzymes, heme oxygenase-1 (HO-1), superoxide dismutase (SOD), and catalase (CAT), leading to the accumulation of ROS in hepatocytes and causing mitochondrial damage and disturbances in energy metabolism [81]. Subsequently, the downregulation of AMPK with the activation of mTOR and its substrate p70 ribosomal S6 kinase (p-S6K) and the phosphorylated S6 ribosomal protein (p-S6) respond to intracellular energy-related abnormalities, which leads to hepatic lipid accumulation in the liver [81]. These results demonstrate that the deleterious metabolic effects produced by CS may be responsible for hepatic steatosis, thus increasing the risk of MASLD.

#### 2.1.4. CS and AMPK Signaling Pathway in Extrahepatic Tissues

At specific sites outside of the liver, smoking activates AMPK, thereby exacerbating the course of MASLD. After CS exposure, nicotine increases insulin resistance by activating the AMPKα2-related signaling pathway [82]. Specifically, nicotine activates AMPKα2 in adipose tissue, which, in turn, phosphorylates MAP kinase phosphatase-1 (MKP1) at serine 334, contributing to its degradation [82]. Following a decrease in MKP1 levels, p38 mitogen-activated protein kinase (p38 MAPK) and c-Jun-NH 2 -terminal kinase (JNK) are activated. JNK phosphorylates insulin receptor substrate 1 (IRS1) at Ser307, leading to the degradation of IRS1 and the inhibition of AKT (protein kinase B), ultimately enhancing lipolysis in adipose tissue and increasing circulating FFA levels [82]. As mentioned earlier, FFAs are one of the main sources of liver steatosis. It is important to note that nicotine accumulates in the human digestive tract following exposure to smoking products and the intensity of AMPKα1 activation due to smoking in the terminal ileum is positively correlated with the amount of nicotine in smokers [83]. The aggregation of nicotine in the ileum induces phosphorylation of Thr172 in AMPKα1 and promotes AMPKα1 activation in a dose-dependent manner. Subsequently, the Ser208/209 sites of SMPD3 are phosphorylated by activating AMPKα1, which in turn inhibits Lys103 ubiquitination on SMPD3, resulting in the inability of SMPD3 to be degraded by the proteasome and maintaining the stability of SMPD3. Overaccumulation of SMPD3 in the ileum increases the synthesis of ceramide, which is a hepatotoxic substance that enhances FFA uptake by hepatocytes and induces insulin resistance and hepatic steatosis [84,85,86]. However, the gut bacterium *Bacteroides xylanisolvens* inhibits the AMPKα/SMPD3/ceramide axis by degrading nicotine in the gut [86]. Therefore, we can confirm that studying the gut–liver axis can enhance our understanding of the relationship between MASLD and systemic metabolism in order to find new treatment options.

To sum up, we hypothesize that the AMPK signaling pathway affected by CS is histologically specific and that it also differentially regulates hepatic lipid metabolism. In addition, the AMPK signaling pathway plays a leading role in the prognostic impact and should be the focus of studies related to CS and MASLD lipid metabolism. In the liver, CS downregulates AMPK, thereby increasing hepatic steatosis. In adipose tissue and the ileum, CS instead upregulates AMPK and produces a host of metabolic problems, which exacerbate the course of MASLD. Therefore, AMPK must be a meaningful therapeutic target. Future studies could pay attention to AMPK-related signaling pathways in order to find more smoking-related reversal factors.

### 2.2. Smoking Perturbs Hepatic Lipid Metabolisms by Influencing Other Signaling Pathways to Promote MASLD

Although the AMPK signaling pathway has emerged as a pivotal molecule in recent investigations exploring the association between CS and MASLD, it is noteworthy that CS exerts its influence on liver steatosis through diverse alternative pathways. For instance, nicotine increases the activity of plasma and hepatic adenosine deaminase (ADA) and XO (xanthine oxidase), as well as the level of uric acid (UA) [87]. The activation of the ADA/XO/UA pathway by nicotine has been demonstrated to inhibit hepatic glycogen production and promote TG accumulation and insulin resistance in the liver, while lithium chloride (LiCl) can effectively suppress this signaling pathway [87]. In serum, elevated levels of triglycerides are an important factor in the induction of hepatic steatosis and hepatic inflammation in MASLD [88]. Mikhail et al. demonstrated that nicotine induced the release of catecholamines, which in turn increased serum triglyceride levels, in adult rats exposed to tobacco smoke from filtered cigarettes, twice a day, for four months [89]. Long-term exposure to environmental tobacco smoke (ETS) reduces high-density lipoprotein cholesterol (HDL-C), which increases the risk of hypertriglyceridemia [90,91]. Moreover, nicotine-induced upregulation of diacylglycerol acyltransferase-1 (DGAT1) facilitates the hepatic conversion of diacylglycerol to triacylglycerol, thereby exacerbating triglyceride dysregulation in the liver [92]. CS exposure also contributes to the progression of MASLD by promoting insulin resistance through the following pathways. Direct exposure to CS leads to a decrease in the insulin concentration in mice, thereby activating the PGC-1α/FOXO1 (Forkhead box proteins O1) pathway, which increases hepatic gluconeogenesis [93]. This glucose metabolism disorder provides a substrate source for hepatic DNL [94]. In human liver L-02 cells, CS extract (CSE) can induce insulin signaling disruption through the inhibition of the IRS-1/Akt signaling pathway and CSE exposure also results in a dose-dependent decrease in glucose uptake and intracellular glycogen levels, which are typical of insulin resistance [95]. Researchers have also found that CS-mediated liver injury and steatohepatitis were accompanied by impaired insulin/insulin growth factor (IGF) signaling, so CS exposure (either first or second hand) may be a cofactor in MASLD [96]. CS can downregulate PPAR-α, thereby inhibiting FA β-oxidation and causing lipid accumulation [97,98,99]. This process can be achieved through the inhibition of the PPAR-α/CPT-1 signaling pathway by nicotine [100,101]. Carnitine O-palmitoyl transferase 1 (CPT-1) is a major regulator controlling the entry of fatty acids into mitochondrion and is an indispensable component in the initiation of FA β-oxidation [102]. In the liver, the CS-induced unfolded protein response (UPR) can cause ER stress, which in turn promotes de novo lipid synthesis and very low-density lipoprotein (VLDL) assembly, leading to hepatic steatosis [92]. Souza et al. demonstrated that nicotine injection in male neonatal mice could trigger the peIF2α/UPR/ER stress pathway in the liver and disrupt lipid homeostasis in the liver [92]. Interestingly, different experiments have found that the downregulation of PPAR-α and the generation of ER stress are sex dependent and that nicotine tends to produce stronger toxic effects in male mice than in female mice [92,99]. Male mice exposed to a nicotine solution tended to be subjected to stronger ER stress than female mice, resulting in more severe hepatic steatosis and more pronounced signs of MASLD [92]. This may be explained by nicotine’s ability to upregulate androgen receptor levels in male mice, thereby enhancing their sensitivity to testosterone [103]. The mRNA levels of the markers of ER stress (Bip, Ire1α, Atf4, and spliced Xbp1) have been reported to be significantly higher in the livers of testosterone-supplemented male rats than in the control group, which demonstrates the ability of testosterone to significantly increase the activity of ER stress in rats [104]. In addition, estrogen is able to activate the ERK/p65 signaling pathway to reduce ER stress [105]. However, the effect of this signaling pathway on CS-induced MAFLD requires further experiments and discussion. It is now well-established that PPAR-α levels are reduced in male mice after CS induction, whereas no consistent changes are observed in female mice [99]. This difference is essential because CS inhibits the activity of SIRT1 and, thus, its downstream target, PPAR-α [99]. The mechanism by which CS exposure specifically inhibits the SIRT1/PPAR-α signaling pathway in male mice should be a key focus for future research, and whether estrogen plays a protective role in regard to this pathway also warrants further investigation. Gender differences may provide novel insights into the development of new therapeutic modalities based on a series of related signaling pathways related to CS/liver lipids in MASLD.

In contrast to NAFLD, MASLD introduces a new subcategory, MASLD with increased alcohol intake (MetALD), which relates to a neglected group of MASLD patients who drink alcohol [9]. Research suggests that patients with nicotine and alcohol dependence are a separate population that requires a great deal of attention from treating physicians [106]. Since CS exposure often occurs in conjunction with alcohol consumption, it is necessary to study the toxicity of their combined effects on the progression and severity of MASLD [107]. Bailey et al. demonstrated that, in terms of hepatic fat accumulation, only 5% of hepatocytes in ethanol-exposed mice alone contained fat droplets, but in the ethanol-plus-ETS group, 50% of hepatocytes in all regions of the liver lobules contained fat [107]. In addition, the combination of nicotine and ethanol more significantly promoted the upregulation of the Plin2 mRNA in the livers of wild-type mice compared to ethanol exposure alone [108]. Plin2 encodes an adipose differentiation-associated protein (ADRP), a marker of fatty liver, and lipid droplets surrounded by ADRP were observed in the hepatocytes of mice with combined nicotine and ethanol exposure after IHC staining, demonstrating that lipid accumulation occurs in the liver of mice [108]. For MetALD, the primary goal of treatment is abstinence from alcohol [109]. However, the effect of giving up drinking on the body’s metabolism is controversial. Because abstinence from alcohol may stimulate the sympathetic nervous system, it can increase blood glucose by producing insulin resistance, thus presenting a risk factor for MASLD [110,111]. Therefore, the treatment of MetALD is a relatively new field and researchers could start with studying the main signaling pathways involved to find the best treatment.

To summarize this section, the increased hepatic lipid accumulation due to CS is the result of a combination of mechanisms. Upregulating hepatic lipid synthesis genes through the abovementioned pathways, disrupting hepatic lipid metabolism, inducing imbalances in body lipid composition, interfering with mitochondrial synthesis and survival, and increasing ER stress are among the processes that lead to the first manifestation of MASLD: hepatic steatosis [112]. In general, preventing liver steatosis caused by CS will be the focus of future drug development to combat MASLD.

## 3. Smoking Facilitates MASH by Activating Hepatocellular Damage

**Figure 2:**
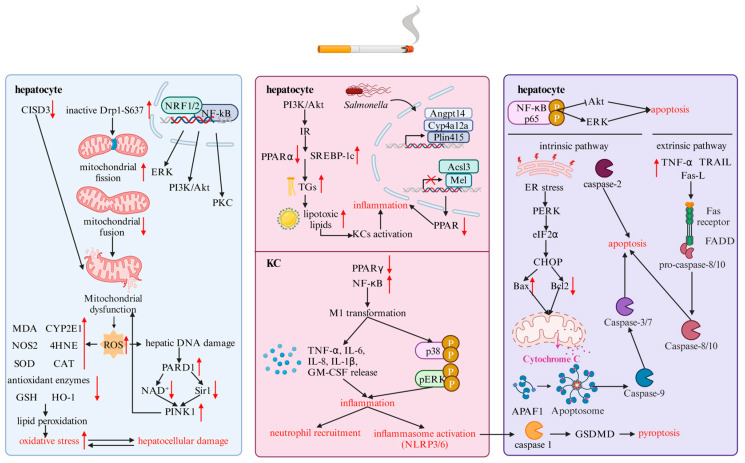
The molecular mechanism of CS-induced hepatocellular damage. CS can trigger the injury and death of hepatocytes through oxidative stress, inflammation, and apoptosis-related signaling pathways. The black arrows represent the progression of molecular mechanism in MASLD. The red arrows represent the positive or negative effect of CS exposure on development and progression of MASH. The red cross mark represents CS exposure can inhibit the transcription of Acsl3 and Mel. Abbreviations: CISD3: CDGSH iron sulfur domain 3; Drp1-S637: dynamin-related protein 1 serine 637; MDA: malondialdehyde; CYP2E1: cytochrome P450, family 2, subfamily E, polypeptide 1; NOS2: nitric oxide synthase 2; 4-HNE: 4-hydroxynonenal; SOD: superoxide dismutase; CAT: catalase; GSH: glutathione; HO-1: heme oxygenase 1; ROS: reactive oxygen species; PARD: programmed death receptor; NAD+: nicotinamide adenine dinucleotide; Sir1: silent information regulator 1; PINK1: PTEN-induced putative kinase; NRF1/2: nuclear respiratory factor 1; NF-κB: nuclear factor-kappa B; ERK: extracellular regulated protein kinase; PI3K/Akt: phosphatidylinositol-3-kinase/protein kinase B; IR: insulin resistance; PKC: protein kinase C; PPARα: peroxisome proliferator-activated receptor α; SREBP1c: sterol regulatory element-binding protein-1c; TGs: triglycerides; KCs: Kupffer cells; SMPD3: sphingomyelin phosphodiesterase 3; AMPKα1: AMP-activated protein kinase; TNF-α: tumor necrosis factor α; IL: interleukin; GM-CSF: granulocyte–macrophage colony-stimulating factor; NLRP3/6: Nod-like receptor protein 3/6; ER stress: endoplasmic reticulum stress; PERK: protein kinase RNA-like ER kinase; eIF2α: eukaryotic initiation factor 2; CHOP: CCAAT–enhancer-binding protein homologous protein; Bcl2: B-cell lymphoma-2; Bax: Bcl-2 associated X protein; APAF1: apoptotic peptidase activating factor-1; GSDMD: gasdermin D; TRAIL: tumor necrosis factor-related apoptosis-inducing ligand; Fas-L: Fas ligand; FADD: Fas-associating protein with a novel death domain. (This figure was created with biorender.com, accessed on 10 December 2024).

The liver belongs to the digestive system and is an indispensable organ that metabolizes more than 40 cigarette-related compounds [113]. Therefore, in the presence of hepatocellular damage, cigarette smoking can increase the liver burden and accelerate the progression of MASLD. Mechanically, smoking can aggravate hepatocellular damage through augmenting the IR, oxidative stress, inflammation, and apoptosis, as depicted in Figure 2. Furthermore, the co-effect of alcohol and smoking can significantly exacerbate oxidative stress, inflammation, and the regeneration of hepatocytes, which may play a more important role in the progression of MASLD than smoking alone [114,115].

### 3.1. Oxidative Stress Signaling Pathway

The excess production of ROS and a deficiency in terms of antioxidants can induce oxidative stress, further aggravating organelle dysfunction, inflammation, and fibrosis of the liver. In smoking conditions, free radicals are generated in large quantities, oxidizing the mitochondrial DNA, proteins, and lipids in hepatocytes, which leads to further hepatocellular damage, inflammation, and cirrhosis, particularly during the tar phase of CS [77,116]. In multiple experimental studies, MASLD models have been made by using the Western diet (WD), which refers to a high-fat or high-fat and glucose diet. Park et al. found that the co-effect of MCS and methionine and a choline-deficient plus high-fat (MCDHF) diet triggered oxidative stress in the liver, by detecting increases in malondialdehyde (MDA), CYP2E1, and NOS2 transcription, and decreases in glutathione (GSH) and HO-1. Apart from oxidative stress markers, they also identified hepatocellular injury, exhibiting elevated ALT, AST, hepatic total cholesterol (TC), and TG in the serum of C57BL/6 mice with steatosis [117]. The concentration of GGT was also found to be higher in MASLD patients with CS exposure, which may heighten the risk of cardiovascular diseases [118]. Several studies have also pointed out that heated tobacco products (HTPs) and e-cigarettes can impair SOD, CAT, and antioxidant enzyme activity and promote lipid peroxidation, except for the abovementioned oxidative stress markers [113,116,119,120,121]. Furthermore, Hasan et al. discovered that 4-HNE, a molecule involved in lipid peroxidation, is upregulated in ENDS and is regarded as a specific marker for oxidative stress [60]. In particular, SOD can produce H_2_O_2_ and ROS [122]. Chen et al. demonstrated that 4-HNE was increasingly expressed in nicotine and cotinine-aggravated alcoholic liver disease mice models, stimulating the effect of *Cdkn1a*-encoded P21, which ultimately impaired the regeneration markers of hepatocytes, including the proliferating cell nuclear antigen (PCNA) and Ki67 [108]. Aside from animal models, the total exposure study (TES) conducted by Liu and colleagues reported that, in the case of adult smokers, the mean level of 8-epi-prostaglandin F2α-, another marker of oxidative stress, was significantly statistically different relative to non-smokers. Additionally, a positive correlation was observed between this marker and the number of cigarettes smoked per day (CPD) [123]. Consequently, through enhancing oxidative stress, CS plays a pivotal role in damaging hepatocytes, which paves the way for fibrosis and the occurrence of HCC in MASLD patients [113]. In addition, Ashakumary and Vijayammal reported that in rats, the combinatory effect of alcohol and nicotine could more significantly impair the activity of SOD, CAT, and glutathione reductase, promote the function of glutathione peroxidase, and enhance the expression of lipid peroxidation products, including malondialdehyde, hydroperoxide, and conjugated dienes, than the separate action of alcohol alone [114]. Furthermore, various studies have revealed that the occurrence and severity of oxidative stress, inflammation, apoptosis, and fibrosis are consistent with the CS exposure time, regardless of the condition of MSCS or third-hand smoke (THS) [122,124,125]. For instance, Neema et al. discovered that, in an in vivo THS exposure system involving mice, accelerated H_2_O_2_ was detected after two months of exposure, and declines in CAT and GPX were measured between four to six months. However, at six months, DNA damage and activated lipid peroxidation did not occur [122]. Kim et al. pointed out that the markers of hepatocellular injury, such as ALT and AST, were higher in early MSCS exposed MCDHF models than in late exposed ones. Nevertheless, the differences in the quantity of such markers were not statistically significant in the CS300 and CS600 experimental groups [124]. Moreover, CS-induced hepatocellular injury also propels oxidative stress by increasing the release of gamma-glutamyl transferase (GGT), which further increases cardiovascular risk in patients with MASLD [118]. Therefore, oxidative stress and hepatocyte damage affect each other bidirectionally in regard to the progression of MASLD.

Furthermore, the detrimental effects of mitochondrial dysfunction-induced excessive ROS production on oxidative stress have also been extensively studied recently. Mitochondrial dysfunction and impairment of the electron transport chain (ETC) and respiratory chain can cause excessive electrons to bind to redundant O_2_, augmenting the formation of ROS. ROS proceeds to lipid peroxidation, ultimately resulting in oxidative stress [122,126]. Conversely, oxidative stress can also facilitate mitochondrial dysfunction, resulting in a vicious cycle and positive feedback in MASLD patients. Wei et al. indicated that, in terms of the co-effects of CS and a high-fat diet (HFD), the CDGSH iron sulfur domain 3 (CISD3) was significantly decreased, which modulates the mitochondrial function and the generation of ROS [127]. In addition, Kanithi et al. also stated that all CS, e-cigarettes, and THS can impel the secretion of ROS. In CS conditions, Drp1-S637 is dephosphorylated to promote mitochondrial fission and impede mitochondrial fusion. It also activates the NF-κB, NRF2, extracellular signal-regulated kinase (ERK), phosphatidylinositol 3-kinase (PI3K)/protein kinase B (Akt), and protein kinase C (PKC) signaling pathways. Regarding the effects of e-cigarettes, high and low concentrations of nicotine produce ROS by boosting mitochondrial swelling and perfusion, respectively [128]. Furthermore, according to Espinoza-Derout et al., the accumulation of ROS can trigger hepatic DNA damage, facilitating the activity of poly (ADP-ribose) polymerases 1 (PARP1). Active PARP1 further reduced NAD^+^ and anti-MASLD SIRT1 and boosted the function of pro-mitochondrial mitophagy PTEN-induced kinase 1 (PINK1) in an apolipoprotein E knockout (ApoE−/−) mouse model of MASLD fed with a WD [129]. Rivera et al. found the same pathological molecular processes present in regard to a combined treatment of nicotine and a high-glucose diet. Moreover, the experiment conducted by Bailey et al. revealed that, apart from the generation of ROS, the production of inducible nitric oxide synthase (iNOS) and reactive nitrogen species (RNS) were much higher in regard to the co-exposure to ETS and ethanol than the individual effect of ETS in male ApoE−/− mice models, which further aggravate oxidative stress, nitrative stress mitochondrial dysfunction, inflammation, and the hepatocellular damage in fatty liver disorders [107]. Consequently, we can infer that MASLD patients exposed to alcohol and smoking may manifest more severe histopathological changes.

### 3.2. Inflammation-Related Signaling Pathway

It has been reported that oxidative stress can boost inflammation [122]. In MASLD, Kupffer cell (KC) activation is crucial for liver inflammation. KC, a resident macrophage in the liver, has a pro-inflammatory M1 phenotype and an anti-inflammatory M2 phenotype, whose activation can induce the release of inflammatory cytokines, the recruitment of neutrophils via chemokine CCL2, and the activation of inflammasomes [130]. Multiple studies have reported that in a mouse model of MASLD fed with the combined treatment of CS or other CS forms and a WD, dramatic levels of secretion of inflammatory cytokines were measured, including tumor necrosis factor α (TNF-α), IL-6, 1L-8, and IL-1β [116,117,121,131,132,133,134]. Zhou et al. indicated that, in response to the co-effect of palmitic acid (PA), LPS, and total particulate matter (TPM) or CS extract (CSE), hepatocellular injury was observed due to the presence of increased ALT and AST, and excessive TNF-α and IL-1β transcription and translation were detected in the co-culture of HCs and KCs [131]. Kim et al. concluded that the downregulation of the IL-1 receptor antagonist (IL-1ra), an anti-inflammatory molecule, may cause inflammation, and the upregulation of TNF-α and IL-1β were not observed in a single culture system, which may reveal the correlation between HCs and KCs [117]. In addition, Frost-Pineda et al. carried out a TES and revealed that the average ALT and AST were not significantly statistically higher in adult smokers compared to non-smokers, whereas the mean ALP was significantly statistically increased in the adult smoker group [135]. Moreover, an experiment conducted by Fouda et al. unveiled that the upregulated expression of pro-inflammatory cytokines generated by a treatment with CS and a HFD in an in vivo MASLD mouse model could enhance the expression of proteins involved in profibrogenic signaling pathways, such as NF-κB and IκB [116]. In addition, similar to exposure time-dependent oxidative stress, the increase in pro-inflammatory cytokines, such as IL-1α, IL-6, the granulocyte–macrophage colony stimulating factor (GM-CSF), and TNF-α, and phosphorylated inflammatory signaling proteins, including p38 and p-ERK, was measured over two months of exposure [122]. However, Azzalini et al. discovered that a long CS exposure time did not upregulate the expression of genes encoding ICAM-1 and TNF-α [125]. Simultaneously, Kim et al. also found that an increase in the SSCS exposure concentration and exposure time did not promote the progression of MASH, by detecting the levels of serum ALT, AST, hepatic TC, and TG in either the MCDHF or control-feeding groups [136]. KC activation and M1/M2 polarization are also modulated by PPARγ [124,134,137]. CS can decrease the activity of PPARγ and promote the phosphorylation of NF-κB, which makes the pro-inflammatory M1 macrophage predominant and augments MASH progression.

Apart from the release of pro-inflammatory cytokines and the phosphorylation of consistent signaling pathways, lipotoxicity and homeostasis of intestinal bacteria also contribute to hepatocellular damage and inflammation in MASLD patients. Lipotoxicity is the most significant step connecting steatosis, inflammation, apoptosis, and fibrosis in the progression of MASLD [138]. However, an excessive amount of synthesized TGs in MASLD patients are lipo-protective, rather than lipotoxic. In the conditions involving lipid accumulation, mitochondrial dysfunction, and increased ROS production, multiple lipotoxic lipids and corresponding metabolites can accumulate in hepatocytes, such as saturated FAs, ceramides, bile acids, glycerophospholipids, and cholesterol, thus resulting in lipotoxicity and hepatocellular damage [138,139,140]. Zhou et al. pointed out that TPM can also aggravate PA-induced cytotoxicity and lipotoxicity, mediated by accelerated TGs, the downregulation of PPAR-α, and the overexpression of SREBP-1c [131]. Furthermore, such exacerbated lipotoxicity can facilitate KC activation and liver inflammation in MASLD patients. Simultaneously, Huang et al. revealed that maternal nicotine exposure (MNE) may result in the stimulation of the PI3K/AKT signaling pathway, which can augment IR through diminishing glucose absorption or impeding glucogenesis in MNE-HFD models [23]. Aggravated IR further downregulates the expression of SREBP-1c and PPARα, eventually paving the way for inflammation and apoptosis in MASLD patients [141]. Other than PA-induced lipotoxicity mentioned above, ceramide also plays a vital role in lipotoxicity. In response to the intervention of nicotine, ceramide, a lipotoxic lipid, is produced extensively in the intestine by phosphorylating AMPKα1 and SMPD3 S208/209, which may impede the degradation of SMPD3 and the progression of MASLD [86,142]. With respect to the contribution of gut bacteria to the progression of MASLD, the key mechanism is an imbalance between beneficial and harmful intestinal bacteria. *Bifidobacterium* and lactic acid bacteria can reduce the nicotine-induced expression of IL-8 and NF-κB, and promote the anti-inflammatory effect of Treg [133]. In contrast, *Salmonella* facilitates the expression of Angptl4, Cyp4a12a, Plin4, and Plin5 genes, and suppresses Acsl3 and Me1 genes, thus boosting the downregulation of PPAR, impairments in lipid metabolism, and inflammation. Nevertheless, *Ligilactobacillus* can counteract this condition [143]. As a consequence, CS may further the inflammation and progression of MASH by promoting the domination of harmful bacteria.

### 3.3. Apoptotic Signaling Pathway

Apoptosis is a critical step in the progression of MASH, whose manifestation is related to the balance between pro- and anti-apoptotic substances and signaling proteins, such as caspase 3, caspase 9, caspase 8, Bax, and Bcl-2 [138,144,145]. Wei et al. discovered that, using a combination of CS and a HFD, cleaved caspase 3 and caspase 9 were accelerated, and anti-apoptotic Bcl2 was reduced in MASLD models. They studied CISD3, which could ameliorate oxidative-induced apoptosis, encountering a CS intervention [127]. Apart from conventional CS, ENDS can also trigger the elevation of caspase 3 and caspase 9 [60]. Apoptosis can also be triggered by CS-induced lipotoxicity. Lipotoxic lipids can stimulate inflammation, disrupt the integrity of hepatocyte cell membranes, release extracellular vesicles, and affect hypoxia [146,147]. Both lipotoxicity-induced apoptosis in hepatocytes and adipose tissue injury can activate the NF-κB signaling pathway, which stimulates the secretion of TNFα and IL-6 [130]. In turn, TNFα can phosphorylate the NF-κB and JNK signaling pathway [130,148]. Consequently, a vicious cycle will be formed that exacerbates the inflammatory response and hepatocyte injury gradually. Azzalini et al. noted that when exposed to CS, NF-κB p65 is phosphorylated, which may cause an alteration in the status of two intracellular signaling pathways: AKT and ERK. AKT is anti-apoptotic, whereas ERK is pro-apoptotic. Consequently, CS exposure can induce the activation of ERK and the dephosphorylation of AKT. Interestingly, in this experiment, caspase 3 and SMAD2, another pro-apoptotic signaling pathway, were not activated [125]. Furthermore, hepatocellular apoptosis was only observed in the co-culture system of HCs and KCs, rather than in the single HC culture MASLD model [117,131]. Similar to exposure- and time-dependent oxidative stress and the inflammatory response, Kim et al. also pointed out that in the first three weeks of being fed an MCDHF diet, exposure to a low concentration of MSCS resulted in a prominent incidence of hepatocyte apoptosis, whereas another model fed with an MCDHF diet and exposed to a high concentration of MSCS during the last three weeks showed a relative decrease [124]. Therefore, we may infer that the damage caused by CS exposure to hepatocytes is more severe in the preliminary stage than in the later stage. Overall, in the context of CS exposure, the molecular mechanism of apoptosis in MASH includes intrinsic and extrinsic pathways, mediated by caspase 3/7 and caspase 8 activation, respectively [138,144,145]. Regarding the intrinsic pathway, lipotoxicity-induced ER stress, mitochondrial dysfunction, dysregulated lysosomal permeability, and JNK activation all contribute to the activation of caspase 3/7 directly, or to the cleaving of procaspase 2 into caspase 2 indirectly [138,144]. In a previous study, the knockout of caspase 2 mitigated MASH molecular changes in mice models [149]. Concerning the extrinsic pathway involved in apoptosis, CS exposure-induced lipotoxicity plays a crucial role in upregulating and activating death receptor 5, apoptosis antigen 1 (Fas), and tumor necrosis factor receptor 1 (TNF-R1) in MASH experimental models, which may induce the activation of caspase 8, ultimately leading to hepatocyte apoptosis [145].

Furthermore, several studies have stated that pyroptosis is also influenced by CS exposure in MASLD models. Su et al. indicated that nicotine could spark the activation of the NLR family pyrin domain containing 3 (NLRP3) and NLRP6 inflammasome, which facilitate the cleavage of pro-caspase 1 into active caspase 1 [150]. Active caspase 1 further promotes the secretion of IL-18 and IL-1β, thereby exacerbating inflammation and activating gasdermin D (GSDMD). Such active GSDMD regulates the pore formation in the plasma membrane of hepatocytes, thus triggering hepatocellular injury during the advancement of MASLD [138,151,152]. Liu et al. found that the effect of procyanidin B2 (PCB2) on pyroptosis-induced hepatocellular death was similar to that of rosiglitazone (RGZ), a selective agonist of PPARγ, showing reduced NLRP3 and inactive GSDMD caused by nicotine. However, in the presence of GW996, an antagonist of PPARγ, PCB2 failed to ameliorate nicotine-caused pyroptosis of hepatocytes in MASLD patients [152]. Moreover, necrosis also comprises MASH-related cell death, but the connection between necrosis and CS exposure has not been elucidated. Necrosis is mediated by receptor-interacting protein 3 (RIP3) and RIP1, enhancing the pore formation effect of mixed lineage kinase domain-like pseudokinase (MLKL). However, the activity of RIP3 is negatively modulated by caspase 8 [138,151].

## 4. Smoking Promotes Liver Fibrosis Through Multiple Pathways to Facilitate MASLD

**Figure 3:**
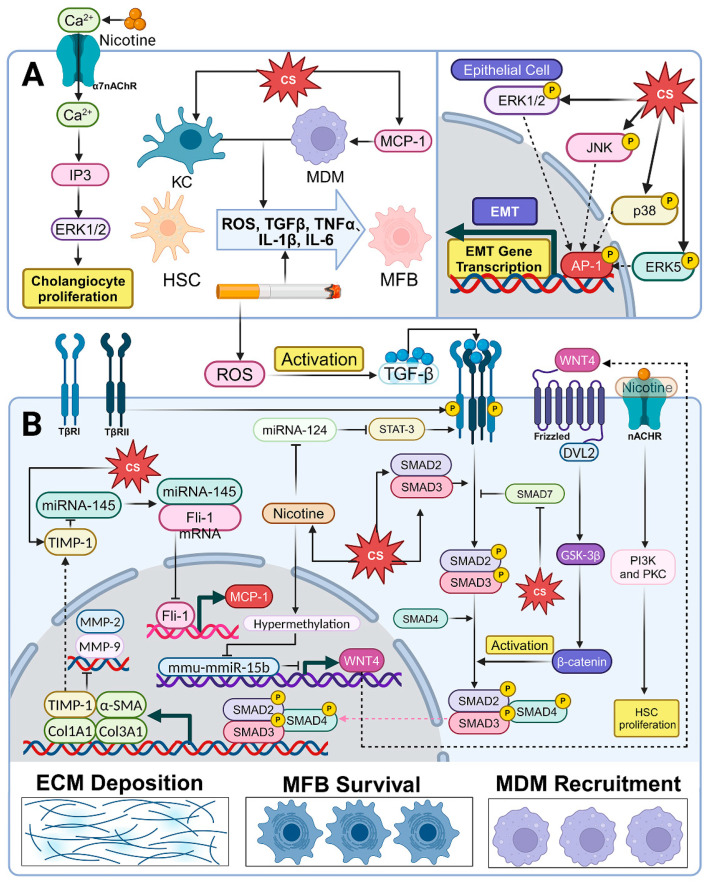
Downstream signaling in regard to the promotion of liver fibrosis by CS. The solid arrows represent ‘facilitation’, the dashed arrows in different colors represent ‘transport’, and the solid ‘T’ lines represent ‘inhibition’. (**A**) Nicotine promotes cholangiocyte proliferation. CS activates macrophages, generates ROS and inflammatory molecules to transform inactive HSCs into active MFBs. Additionally, the CS/MAPK/AP-1 signaling pathway in hepatic epithelial cells can promote EMT, which is a potential source of MFB. (**B**) CS-induced signaling pathway in HSCs. CS can trigger various downstream signaling pathways to activate collagen production, promote HSC proliferation and survival, and stimulate the recruitment of MDMs to continuously induce liver fibrosis. Abbreviations: CS: cigarette smoke; KC: Kupffer cell; MDM: monocyte-derived macrophages; MCP-1: monocyte chemoattractant protein-1; HSC: hepatic satellite cell; MFB: myofibroblasts, ROS: reactive oxygen species; TGF-β: transforming growth factor-β; TNF-α: tumor necrosis factor-α; IL-1β: interleukin-1β; IL-6: interleukin-6; EMT: epithelial–mesenchymal transition; AP-1: activator protein-1; TβRI: TGF-β type I receptor; TβRII: TGF-β type II receptor; nAChR: neuronal nicotinic acetylcholine receptor; DVL-2: disheveled 2; STAT-3: signal transducer and activator of transcription 3; TIMP-1: metalloproteinase-1; MMP: matrix metalloproteinase; Fli-1: friend leukemia virus integration-1; α-SMA: α-smooth muscle actin; Col1A1: collagen type I α1 chain; Col3A1: collagen type III α1 chain; GSK-3β: glycogen synthase kinase-3β; PI3K: phosphatidylinositol-3-kinase; PKC: protein kinase C. (This figure was created with biorender.com, accessed on 3 February 2025).

Smoking is a risk factor for liver fibrosis in MASLD. A set of bivariate analyses, based on human liver biopsies, showed that MASLD patients with a history of smoking were more likely to develop advanced liver fibrosis [153]. An iterative nested model study, using likelihood ratio testing, involving 989 patients in the US, similarly demonstrated that smoking is one of the key determinants of the progression of liver fibrosis: smokers were 89% more likely than non-smokers to have higher odds of progression per year after MASH diagnosis [154]. In addition, a recent set of multiple linear regression analyses, involving 1433 US adolescents, noted a good dose–response correlation between serum cotinine (a metabolite of nicotine) and liver fibrosis (*p* < 0.001) [155]. Mary et al. also confirmed that the proportion of smokers was significantly higher in liver fibrosis-positive MASLD patients than in the liver fibrosis-negative group [156]. A cross-validated retrospective assessment concluded that lifetime tobacco consumption (≥10 packs/year) was significantly associated with advanced liver fibrosis at the time of consultation, as exposure to CS may accelerate the progression of liver fibrosis in those exposed to it [157]. In a bivariate logistic regression analysis of 598 subjects, Balogun et al. found that patients with T2DM who used tobacco were 2.4 times more likely than the controls to develop advanced fibrosis [158]. However, another set of clinical investigations did not find a direct correlation between smoking and liver fibrosis [159]. This variation in the results may be related to unknown confounding variables, different sample sizes, and non-uniform criteria for judging smoking status [155]. Indeed, many experimental studies involving animal models have visually demonstrated, at the molecular level, that smoking promotes liver fibrosis. In MASLD, sequential occurrences of lipotoxicity, oxidative stress, ER stress, inflammation, and apoptosis in hepatocytes lead to the activation of hepatic regeneration and fibrogenesis, deposition of the extracellular matrix (ECM), and the promotion of hepatic fibrosis and cirrhosis [139]. CS is involved in the above processes and promotes liver fibrosis in several ways (Figure 3).

### 4.1. CS Triggers Cellular and Molecular Changes

The activation of HSCs is a crucial step in the progression of liver fibrosis, as it facilitates their transformation into MFBs, which are responsible for the excessive production of ECM components, such as collagen fibers [160] CS-induced production of ROS, TGFβ, TNF-α, IL-1β, and IL-6 can directly transform HSCs into MFBs [27,119,161,162,163]. Moreover, it has been established that monocyte-derived macrophages (MDMs) and KCs are key modulators during liver fibrosis, because they can synthesize the aforementioned fibrosis-inducing factors to activate HSCs [164,165]. A recent study identified upregulated levels of monocyte chemotactic peptide-1 (MCP-1) in nicotine-induced liver fibrosis model rats [166]. MCP-1 is a cytokine that has a recruitment effect on MDMs, a process that activates proinflammatory M1 phenotypic changes in MDMs [167]. In addition, in the liver, the exposure to the smoke from four cigarettes per day (five days per week) for 14 weeks was found to facilitate the mRNA expression of F4/80 (a marker for MDMs) and CD68 (a marker for KCs), which increased approximately three-fold compared to the controls groups, demonstrating that CS is a direct inducer of hepatic inflammatory infiltration [116].

Moreover, nicotine can also enhance the proliferation of HSCs, by stimulating the nAChR/PI3K/PKC signaling pathway to promote fibrogenesis in MASH patients [168]. Conversely, mecamylamine, a neuronal nicotinic acetylcholine receptor (nAChR) antagonist, can impede nicotine-induced HSC proliferation [168].

HSCs are not the only source of MFBs [169]. One theory suggests that hepatic epithelial cells (including hepatocytes and cholangiocytes) can also be a direct source of MFBs through the epithelial–mesenchymal transition (EMT) [170]. After assessing the effect of chronic CS exposure on mouse liver EMT using qPCR, Chen et al. proved the occurrence of the upregulation of the hepatic EMT-related mRNA, E-cadherin (CDH1), α-smooth muscle actin (α-SMA), vimentin (VIM), fibronectin (FN), and Twist [163]. Additionally, Liang et al. confirmed a CS-induced EMT phenomenon in the livers of BLAB/c mice, characterized by the downregulation of hepatic epithelial cell markers (E-calmodulin and ZO-1) and the upregulation of mesenchymal cell markers (vimentin and N-calmodulin) [171]. We can, therefore, identify CS as an inducer of EMT in the liver. CS-related hepatic EMT is dependent on the mitogen-activated protein kinase (MAPK)/AP-1 signaling pathway: through the activation of the ERK1/2, JNK, p38, and ERK5 pathways, transcription factor-activating protein-1 (AP-1) is phosphorylated in the liver to activate and, thereby, induce EMT-related gene expression [171]. Therefore, the development of drugs targeting the MAPK signaling pathway and, thereby, inhibiting CS-induced EMT is an effective approach for the treatment of MASLD. However, whether smoking-induced EMT increases the levels of MFBs in the liver needs further exploration. The current results only support our inference that CS-induced EMT could be a potential source of liver fibrosis.

The interaction between ETS and alcohol accelerates the progression of hepatic fibrosis, as the combined exposure to ethanol and ETS increased the α-SMA levels, a classic marker of fibrosis, by 65% and the liver showed increased collagen staining, compared to the ETS only group [107]. The prevailing perspective posits that the proliferation of cholangiocytes within intrahepatic/extrahepatic bile ducts is the instigating factor for fibrogenesis during chronic liver injury [172]. Nicotine treatment can increase the expression of α7nAChR on the surface of normal rat intrahepatic cholangiocyte (NRIC) cultures in order to increase the intracellular calcium concentration [172]. This stimulates the Ca^2+^/IP3/ERK1/2 pathway and induces NRIC proliferation in a dose-dependent manner within 48 h [172]. Therefore, investigating the functionality of nAChRs represents a critical objective in addressing CS/MASLD, with smoking cessation undeniably serving as the most direct and simplistic therapeutic approach to impeding liver fibrosis progression.

### 4.2. TGF-β/SMAD Signaling Pathway

TGF-β is a central regulator in driving the development of liver fibrosis during MASH [173,174]. It promotes the activation of HSCs, the maintenance of the MFB phenotype, and the synthesis of ECM through the TGF-β/SMAD3 signaling pathway [175]. Inactivated TGF-β is first converted into its active form by ROS [176]. Subsequently, activated TGF-β binds to the TGF-β type II receptor (TβRII) to initiate signaling, which, in turn, recruits the binding of the TGF-β type I receptor (TβRI) to form heterotetramers [170]. During this process, TβRII phosphorylates serine and/or threonine residues in the Gly-Ser (GS)-rich structural domains in the near membrane of TβRI, leading to the conformational activation of TβRI [177]. Notably, ROS similarly enhance TβRI activation [178]. The activation of TβRI enables it to phosphorylate its substrates, SMAD2 and SMAD3 [179]. Next, phosphorylated SMAD2/SMAD3 binds to SMAD4 to form a transcriptional complex [180], which subsequently translocates to the nucleus to regulate the expression of target genes in MFBs, including collagens (COL1A1, COL3A1, etc.), TIMP-1, and α-SMA [181,182]. These genes are major markers of stress fiber formation and increased tissue stiffness during hepatic fibrosis [183] and their upregulation implies increased ECM deposition. In regard to the TGF-β signaling pathway, SMAD7 can inhibit the activation of SMAD2/SMAD3 by competing with SMAD2/SMAD3 for the binding of TβRI and, thus, downstream signaling is disrupted [170]. Therefore, SMAD7 consistently plays a protective role in liver fibrosis [184,185]. Past studies have shown that SMAD3 is the key progenitor in the induction of liver fibrosis, although both SMAD2 and SMAD3 are substrates that are significantly activated in liver fibrosis [186,187]. This is because SMAD3 can bind directly to DNA sequences that regulate the expression of multiple collagen genes and fibrosis markers [163,179], thereby directing the progression of liver fibrosis. This implies that the next step in studying the CS/TGF-β/SMAD signaling pathway could focus on lowering the SMAD3 levels and elevating the SMAD7 levels.

The TGF-β signaling pathway has long been a hotspot for investigating the intrinsic link between CS and liver fibrosis in MASLD. TGF-β and ROS are significantly upregulated in nicotine-treated livers and lead to significant thickening and massive collagen deposition in the hepatic blood sinusoidal and confluent regions [188]. As the nicotine concentration increases (from 10 pM, 10 nM to 10 μM), the level of TGF-β shows a step-wise increase [168]. Furthermore, researchers have successfully identified the upregulation of SMAD2 and SMAD3, as well as the downregulation of SMAD7, in the liver of mice following exposure to CS [125,163]. Interestingly, CS can indirectly stimulate the TGF-β/SMAD signaling pathway by affecting epigenetic modifications. In a liver fibrosis model of rats receiving nicotine administration, the level of the anti-inflammatory miRNA-124 was suppressed by nicotine, whereas the expression of its specific target STAT-3 was upregulated during the fibrotic process [166]. STAT-3/TGF-β facilitates HSC activation and hepatic ECM production [189]. In addition, paternal exposure to nicotine downregulates microRNA mmu-mmiR-15b expression by enhancing CpG hypermethylation of its DNA in spermatozoa and, then, this epigenetic alteration is imprinted on the offspring’s liver [190]. The downregulation of mmu-mmiR-15b increases the expression of the target gene, WNT4, in HSCs [190]. WNT4 binds to the GPCR receptor Frizzled (Fz), leading to the activation of the DVL2/GSK-3β/β-catenin pathway in HSCs, thereby initiating the TGF-β/SMAD signaling pathway [190]. This implies a strong association between CS and the initiation and progression of liver fibrosis, through multiple mechanisms. Further investigation is warranted to explore the impact of smoking on TGF-β expression and elucidate the underlying signaling pathways.

### 4.3. TIMP-1 Signaling Pathway

Multiple groups of studies have demonstrated the upregulation of the tissue inhibitor of metalloproteinase-1 (TIMP-1) in liver tissues after CS exposure [117]. TIMP-1 is an HSC-released glycoprotein inhibitor and its main function is to inhibit the activities of matrix metalloproteinase (MMP), thereby inhibiting ECM degradation [191,192,193]. In addition, TIMP-1 can prevent apoptosis of MFBs by inhibiting MMP activity [194]. Therefore, TIMP-1 upregulation is often considered a risk factor for liver fibrosis. According to previous reports, the upregulation of TIMP-1 and an increase in collagens tend to co-exist in liver tissues after CS exposure [117,125,141,161]. At the same time, the nicotine exposure-induced upregulation of TIMP-1 is accompanied by the downregulation of MMP-2 and MMP-9 in liver tissue [141]. Surprisingly, a recent study found that the upregulation of TIMP-1 in MFBs also had immunological effects. TIMP-1 downregulates the expression of miRNA-145, an miRNA that binds directly to the 3′UTR of the mRNA of the transcription factor, Fli-1 [195]. The downregulation of miRNA-145 activates the Fli1/MCP-1 pathway, thus increasing the recruitment of MDMs and shaping the pro-inflammatory immune microenvironment in the liver, exacerbating liver fibrosis [196]. Future drug studies targeting the regulation of TIMP/MMP homeostasis could serve as a promising approach for addressing liver fibrosis in CS-induced MASLD.

## 5. Modulators Capable of Ameliorating Smoking-Induced Exacerbation of MASLD

Recently, the use of CS-assisted induction of MAFLD in experimental models has become a more reliable approach. Savari et al. developed a classical MASH model after feeding mice with a WD (rich in fat, fructose, and cholesterol) [132]. On this basis, the mice treated with CS plus a WD showed a stronger MASH profile, specifically in the form of significantly elevated levels of liver injury markers (AST, ALT, and ALP) and inflammatory markers (TNF-α), and the H&E stained liver sections showed more severe hepatic fat accumulation, hepatocyte swelling, and inflammatory infiltration [132]. This demonstrates that smoking significantly exacerbates the condition on top of the pre-existing MASLD and that providing specific interventions and treatments for smoking-induced MASLD is a necessity. As mentioned above, maintaining a healthy lifestyle remains the current first line of treatment for MASLD [197]. Therefore, in response to the current findings, we review the chemicals with protective effects on smoking-induced MASLD and provide recommendations for lifestyle improvements (Table 1).

### 5.1. Lycopene

Lycopene is a non-provitamin A carotenoid [203], known for its wide distribution in tomatoes (*Solanum lycopersicum*) and related products [204]. Recently, several studies have demonstrated its effectiveness in relieving MASLD triggered by CS exposure.

Moreover, 4-(N-methyl-N-nitrosamino)-1-(3-pyridyl)-1-butanone (NNK) is one of the specific carcinogens in tobacco smoke [205]. In their study, Aizawa et al. directly induced MASH in a model in ferrets via the intraperitoneal (ip) injection of NNK (50 mg/kg BW, once a month for four months), which resulted in severe inflammatory cell infiltration, hepatic fat accumulation, hepatocellular ballooning-like degeneration, and the increased expression of injurious NF-κB, CYP2E1 [198]. They then explored whether lycopene had a therapeutic effect on the NKK-induced MASH ferret model. After supplementation with dietary lycopene (2.2 and 6.6 mg/kg BW/day, respectively) for 26 weeks, the researchers found that lycopene can mitigate NKK-induced MASH in a dose-dependent manner by downregulating CYP2E1 and inflammatory NF-κB [198]. Not coincidentally, another study successfully induced MASH in a model in ferrets through co-treatment with NKK and CS exposure, confirming that the mRNA levels of the fibrosis markers, COL1A1, TGF-β, and TIMP-1, were downregulated by dietary lycopene and the hepatic inflammation grade was also reduced [161]. Interestingly, in the MASH model induced by NKK and CS, the level of the oxidative cleavage enzyme β-carotene-9′,10′-oxygenase (BCO2), which can cleave carotenoids, was also upregulated [161]. In essence, the downregulation of BCO2 is capable of inhibiting the expression of the enzymes and/or proteins involved in fatty acid β-oxidation, the tricarboxylic acid cycle, and the electron transport chain, with the subsequent generation of oxidative stress that increases the amount of ROS [206]. In turn, excess ROS downregulate mitochondrial SOD2, CAT, and GPX, which may be a key step in smoke-induced MASH [207,208,209]. Unsurprisingly, we observed the downregulation of this group of enzymes in the liver after CS and NKK exposure, which was reversed by lycopene [161]. Thus, lycopene may have promising effects in CS-induced MASLD treatments in terms of anti-cellular damage, anti-fibrosis, and anti-oxidation.

Given that maintaining good lifestyle habits remains the dominant approach to controlling the progression of MASLD, we recommend that patients with smoking-related MASLD increase their daily intake of carotenoid-rich vegetables, such as tomatoes, and quit smoking, thereby slowing down the progression of the disease.

### 5.2. Curcumin

Curcumin makes up 3–4% of the turmeric composition of *Curcuma longa* [210], and has anti-inflammatory, cholesterol-lowering, and antioxidant properties [211]. Hepatotoxicity due to nicotine in tobacco smoke has emerged as a risk factor for MASLD [127]. This conclusion was reaffirmed by Salahshoor et al., who showed that a single daily intraperitoneal injection of nicotine (2.5 mg/kg) in mice for four weeks induced MASLD-associated hepatic injury, i.e., a decrease in liver weight and an increase in the mean diameter of the hepatocytes, the hepatic central vein, hepatic enzyme levels, and serum nitric oxide levels [199]. The study also found that an ip injection of curcumin (10, 30, and 60 mg/kg) significantly reduced hepatotoxicity and oxidative stress due to NO-generated hydroxyl radicals, by lowering the nitric oxide (NO) concentrations in mice injected intraperitoneally with nicotine [199]. In addition, curcumin effectively inhibited hepatic injury in nicotine-injected mice [199]. In another experiment, Liang et al. found that the EMT-related MAPK/AP-1 gene was significantly upregulated in the liver of mice exposed to tobacco smoke for six hours per day, for 12 consecutive weeks [171]. As described in Section 4, EMT is a risk factor for liver fibrosis in the later stages of MASLD and a modulator for the development of cancer in MASLD [212]. Notably, Liang et al. found that curcumin (50 or 100 mg/kg body weight per day) was able to significantly slow down the progression of EMT by inhibiting CS exposure-induced activation of the ERK1/2/AP-1 and JNK/AP-1 signaling pathways in mouse liver [171]. Another set of mouse experiments (80 mg/kg orally for 22 weeks) with the curcumin analog, BDMCA, demonstrated that BDMCA could restore nicotine subcutaneous injection-induced levels of cholesterol, triglycerides (TGs), phospholipids (PLs), and FFAs in plasma and tissues (liver, kidneys, etc.). Therefore, curcumin and its analogs may present advantages in regard to anti-MASLD drug development [200].

### 5.3. Epigallocatechin-3-gallate (EGCG)

Epigallocatechin-3-gallate (EGCG) is the most abundant active catechin in green tea (*Camellia sinensis*) [213]. Recent studies have demonstrated that EGCG has a restorative effect on the abnormal tissue structure changes caused by CS. Chen et al. exposed rats to the smoke of one cigarette per rat, once a day, in an exposure chamber (45 × 25 × 20 cm with two compartments) for 90 days, which subsequently induced hepatic EMT, local inflammation, oxidative stress, and activation of the TGF-β1 signaling pathway, which are typical of MASLD and have been described in detail above [163]. Subsequently, rats were manipulated with EGCG by gavage (100 and 50 mg/kg EGCG for 90 days) [163]. EGCG was able to downregulate the levels of TGF-β/SMAD3 after CS exposure, while elevating the levels of SMAD-7, thereby inhibiting the activity of the fibrogenic pathway and reducing the mRNA level of Col1A1 and Col3A1 [163]. Interestingly, the levels of EMT markers (Twist, VIM, α-SMA, FN, and CDH1), lipid peroxidation markers (MDA), and inflammatory molecules (TNF-α and IL-1β) were reduced, whereas the levels of antioxidant enzymes (SOD and GPX) and non-enzyme antioxidants (GSH) were elevated [163].

However, EGCG is not able to completely reverse the liver damage caused by CS. Al-Awaida et al. exposed mice to water-pipe smoke for 90 days and subsequently observed characteristic changes in MASLD in the mice: inflammation in the hepatic portal region and reduced levels of antioxidant enzymes [201]. After treating liver tissues exposed to smoke with EGCG, no significant change in the overall expression was observed, even though there were slight increases in the antioxidant genes, CAT, GPXI, MT-I, MT-II, SOD-I, SOD-II, and SOD-III [201]. The levels of antioxidant genes could not be restored to those of the control group after EGCG treatment [201]. This proves that the damage to the liver caused by smoking is difficult to reverse and that EGCG can only provide some relief. Therefore, we still consider smoking cessation as the primary therapeutic recommendation; in addition to reasonable intake of green tea products as an effective way to hinder the development of MASLD.

### 5.4. Caffeine

Caffeine (1,3,7-trimethylxanthine) is an adenosine receptor antagonist [214], which is a functional substance naturally occurring in certain products, such as coffee, tea, and cocoa beans [215]. It is less well-known that caffeine intake has a protective effect against MASLD [216,217,218,219]. Lu et al. developed a mouse model of MASLD via the ip injection of nicotine plus a (HFD): large numbers of lipid droplets were observed in the livers of both the HFD-alone group and the HFD + nicotine group, but lipid droplets were more abundant in the HFD + nicotine group, with the occurrence of hepatic inflammation and injury, oxidative stress, and steatosis [100]. However, this experiment also proved that caffeine exerted anti-inflammatory effects by downregulating the pro-inflammatory cytokines, IL-1β, IL-6, and TNF-α. Moreover, RT-PCR detected a downregulation in the expression of the lipogenesis genes, ACC, FAS, and SREBP-1c, after caffeine treatment [100]. In addition, caffeine mitigated nicotine-induced steatosis in hepatic tissues by upregulating the PPAR-α/CPT-1 pathway [100]. These results demonstrate the excellent effect of caffeine in reversing the deterioration of nicotine-induced MASLD, from both microscopic and macroscopic perspectives.

We are not advocating that people consume caffeine uncontrollably and as much as possible because excessive caffeine intake can lead to negative effects, such as increased anxiety levels [220], decreased sleep quality [221], accelerated bone loss [222], and increased risk of miscarriage and preterm birth during pregnancy [223]. Therefore, to maintain a good and healthy lifestyle and to reduce the aggravation of MASLD due to smoking addiction, our advice is to consume coffee and tea in moderation, as this does not have long-term adverse health effects [223].

### 5.5. Baccharis Trimer (Less.) DC

*Baccharis trimer* (Less.) DC is a perennial subshrub belonging to the family Asteraceae, originating from South America and mostly found in southern Brazil [224]. *Baccharis trimer* exerts hepatoprotective, lipid-lowering, and antioxidant effects that are mainly dependent on the major compounds in its aboveground parts: flavonoids, terpenes, and chlorogenic acid [202]. In a previous study, rats were fed a diet enriched with 0.5% cholesterol and exposed to CS (nine cigarettes per day, five days per week) over a period of four weeks and this method successfully induced MASLD [202]. Then, the rats received oral treatments involving a B. trimera extract, at doses of 30, 100, and 300 mg/kg [202]. The results of the experiment indicated that the *Baccharis trimer* extract reduced oxidative stress in the liver by lowering the levels of SOD and CAT in the liver, thereby reducing liver damage caused by free radical synthesis [202]. In addition, the *Baccharis trimer* extract reversed CS-induced elevation of hepatic cholesterol and TG levels, and increased fecal excretion of cholesterol related to stimulated liver regeneration [202]. Therefore, future research on hepatoprotective drugs can be linked to the basic research on *Baccharis trimer* extracts in order to utilize the advantages of natural herbs with low toxicity and related effectiveness.

### 5.6. Alpha-Lipoic Acid

Alpha-lipoic acid (ALA) is an organosulfur constituent with strong antioxidant properties, sources of which include meats, vegetables, and fruits [225,226]. Reis et al. confirmed that ALA may be a promising therapeutic agent for MASLD caused by tobacco smoke exposure, because ALA therapy reduces hepatocyte toxicity by decreasing free radicals, ROS, and reactive nitrogen species (RNS) in models of MASLD induced by chronic smoking [119]. In addition, ALA alleviates CS-induced mitochondrial dysfunction in the liver by increasing the levels of GSH and SOD, as ALA is a cofactor for some key mitochondrial enzymes and stimulates the activity of enzymes involved in GSH synthase and other antioxidant enzymes [119]. The levels of IL-6 are also significantly downregulated. Interestingly, it has also been observed that ALA treatment significantly reduced the serum levels of ALT and AST after smoking, suggesting that ALA is capable of repairing CS-induced liver cell necrosis [227].

These findings reaffirm that the combination of a rational diet and smoking cessation is the main approach to alleviating MASLD. Future drug development could also look at the protective effects of ALA, leading to the proposal of an effective drug formulation.

## 6. Conclusions

Unhealthy lifestyles, such as a HFD, sedentary behavior, and smoking, play significant roles in promoting the development of MASLD. Owing to the absence of effective pharmacological interventions for MASLD, lifestyle modifications remain the primary clinical approach. It is noteworthy that numerous experiments have demonstrated the significant promotion of MASLD deterioration due to smoking in both general populations and model organisms. However, a comprehensive summary of the specific molecular mechanisms involved remains elusive. Therefore, this article reviewed the signaling pathways according to which different types of CS exposure patterns affect the progression of MASLD and further explores substances that may reverse the deterioration of MASLD caused by CS in order to find potential therapeutic targets. The intricate interplay between CS and various cellular components in MASLD has been documented. At the same time, due to the importance of lifestyle modifications in the treatment of MASLD, it is essential to understand the role of CS in the progression of MASLD globally. The present study revealed that CS and its detrimental constituents significantly heighten the susceptibility to MASLD, primarily through perturbations in lipid metabolism, the facilitation of hepatocyte injury and apoptosis, and the induction of liver fibrosis. Specifically, CS promotes de novo lipid synthesis, insulin resistance, oxidative stress, inflammatory cell recruitment, cytokine release, collagen synthesis and deposition, the activation and phenotype maintenance of HSCs, and EMT, through multiple signaling pathways. In addition, normal lipid metabolism and ECM degradation are also inhibited by CS, leading to the manifestation of histological characteristics associated with steatosis and fibrosis in the liver. However, recent research is encouraging, as lycopene, curcumin, caffeine, EGCG, Baccharis trimer (Less.) DC, and ALA have been demonstrated, in detail, to ameliorate liver damage caused by CS. This implies that the incorporation of smoking cessation, alongside other favorable dietary practices, has significant implications for managing the condition of MASLD. The current state of knowledge regarding CS and MASLD necessitates further comprehensive investigations in terms of both basic and clinical research, while the exploration of potential therapeutic agents for disease reversal remains limited. Future research on MASLD should prioritize investigating the impact of various lifestyle combinations, thereby encouraging patients to enhance all aspects of their lifestyle habits to prevent the occurrence and progression of chronic diseases. In conclusion, the signaling pathways summarized in this study could be potential targets for future treatments of MASLD, and individuals at risk should be encouraged to quit smoking and adopt a balanced dietary regimen. In the future, our research will focus on animal experiments and clinical trials in order to find more feasible treatment methods.

## 7. Perspectives

With respect to the development and progression of MASLD, CS is increasingly acknowledged as a major risk factor because it affects multiple key pathways in the pathogenesis of MASLD, MASH, cirrhosis, and HCC. Future research focusing on random controlled trials or clinical trials, the underlying mechanisms, and promising treatment strategies for CS-aggravated MASLD will pave the way for more effective prevention and therapy options. Regarding long-term prospective studies, recent studies are mainly cross-sectional, longitudinal, or animal-based studies, revealing that CS exposure may aggravate MASLD and promote the development of cirrhosis and HCC via their interference with lipid metabolism, oxidative stress, mitochondrial dysfunction, ER stress, and fibrosis, which may be limited in terms of its applicability to human health. As a consequence, prospective studies aimed at exploring the relationship between the duration and intensity of CS exposure and the extent of hepatocellular injury can emphasize the importance of smoking cessation in augmenting lipid accumulation, liver inflammation, and fibrosis in MASLD patients. Moreover, other corresponding factors, such as diet, alcohol consumption, and genetic predispositions, should be considered in terms of the effect of CS on MASLD. Epigenetic mechanisms also play an indispensable role in the susceptibility and pathogenesis of CS-exacerbated MASLD. Recent research has reported that CS can trigger various epigenetic changes, contributing to MASLD development. Such changes, including DNA methylation alterations, histone modifications, and changes in non-coding RNA expression, can affect gene expression involved in dyslipidemia and liver inflammation in MASLD patients. Investigating how these modifications influence the expression of crucial metabolic regulators, such as SREBPs and AMPK, could provide insights into the molecular underpinnings of MASLD in patients with a history of smoking. Therefore, future studies elucidating specific epigenetic modifications caused by CS can facilitate the investigation of novel epigenetic therapies targeting these pathways. Furthermore, targeted interventions for smokers with MASLD are also pivotal. Current lifestyle modification strategies emphasize smoking cessation, alongside dietary changes and physical activity. Nevertheless, adherence in regard to these interventions is poor. Consequently, to improve adherence among smokers with MASLD, future research should explore tailored interventions and incorporate them with behavioral science principles. Personalized counseling sessions should focus on overcoming specific obstacles to quitting smoking, while also incorporating pharmacological aids designed to reduce cravings and support liver health. Additionally, pharmacological treatments targeting the mechanisms by which CS exacerbates MASLD could be developed. For instance, nAChR antagonists have shown promise in preclinical studies for reversing nicotine-induced liver injury. Developing such agents in clinical trials can provide new therapeutic avenues for managing MASLD in smokers. In conclusion, future research targeting CS-aggravated MASLD should prioritize long-term prospective studies to establish causal correlations, investigate the epigenetic mechanisms underlying disease progression, and develop novel targeted therapeutics. Ultimately, through future prospective investigations, researchers can significantly increase their understanding of the complicated molecular mechanisms and therapeutic strategies available for smokers at risk of MASLD.

## Figures and Tables

**Table 1:** Natural products that can alleviate MASLD affected by cigarette smoke.

Substance	Chemical Structure	Function	Reference
Lycopene	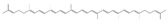	· Downregulation of CYP2E1 and NF-κB to inhibit NKK-induced liver injury. · Inhibition of COL1A1, TGF-β, and TIMP-1 expression to inhibit fibrogenesis. · Reduces oxidative stress in the liver.	[161,198]
Curcumin	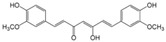	· Reduces the nitric oxide (NO) level to reduce nicotine-induced oxidative stress. · Inhibition of ERK1/2-AP-1 and JNK/AP-1 signaling pathway-related EMT. · Curcumin analogue BDMCA reduces hepatic lipid accumulation.	[171,199,200]
Epigallocatechin-3-gallate (EGCG)	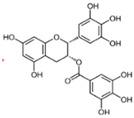	· Inhibition of TGF-β/SMAD signaling pathway reduces fibrogenesis. · Inhibition of EMT by inhibiting the synthesis of the relevant markers. · Anti-inflammatory, anti-oxidative stress. · Reduces pro-inflammatory cytokines IL-1β, IL-6, and TNF-α.	[163,201]
Caffeine	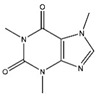	· Inhibition of ACC, FAS, and SREBP-1c to inhibit de novo lipid synthesis. · Upregulation of PPAR-α and CPT-1 to promote lipid metabolism.	[100]
Alpha-lipoic acid	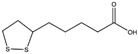	· Reduces liver damage caused by chronic smoking by reducing free radicals, ROS, RNS, and IL-6. · Relieves smoking-induced mitochondrial dysfunction by increasing levels of GSH and SOD.	[119]
Baccharis trimer (Less.) DC	------	· Reduces oxidative stress in the liver by decreasing CS exposure-induced SOD and catalase levels in the liver. · Reduces CS-induced hepatic lipid accumulation and promotes cholesterol excretion.	[202]

## Data Availability

Not applicable.

## References

[1] Song S.J., Lai J.C., Wong G.L., Wong V.W., Yip T.C. (2024). Can we use old NAFLD data under the new MASLD definition?. J. Hepatol..

[2] Aghemo A., Alekseeva O.P., Angelico F., Bakulin I.G., Bakulina N.V., Bordin D., Bueverov A.O., Drapkina O.M., Gillessen A., Kagarmanova E.M. (2022). Role of silymarin as antioxidant in clinical management of chronic liver diseases: A narrative review. Ann. Med..

[3] Huang D.Q., El-Serag H.B., Loomba R. (2021). Global epidemiology of NAFLD-related HCC: Trends, predictions, risk factors and prevention. Nat. Rev. Gastroenterol. Hepatol..

[4] Jahn D., Kircher S., Hermanns H.M., Geier A. (2019). Animal models of NAFLD from a hepatologist’s point of view. Biochim. Biophys. Acta Mol. Basis Dis..

[5] Khamphaya T., Chukijrungroat N., Saengsirisuwan V., Mitchell-Richards K.A., Robert M.E., Mennone A., Ananthanarayanan M., Nathanson M.H., Weerachayaphorn J. (2018). Nonalcoholic fatty liver disease impairs expression of the type II inositol 1,4,5-trisphosphate receptor. Hepatology.

[6] Calzadilla Bertot L., Adams L.A. (2016). The Natural Course of Non-Alcoholic Fatty Liver Disease. Int. J. Mol. Sci..

[7] Targher G., Byrne C.D., Tilg H. (2024). MASLD: A systemic metabolic disorder with cardiovascular and malignant complications. Gut.

[8] Yoon E.L., Jun D.W. (2023). Waiting for the changes after the adoption of steatotic liver disease. Clin. Mol. Hepatol..

[9] Kim G.A., Moon J.H., Kim W. (2023). Critical appraisal of metabolic dysfunction-associated steatotic liver disease: Implication of Janus-faced modernity. Clin. Mol. Hepatol..

[10] Chalasani N., Younossi Z., Lavine J.E., Charlton M., Cusi K., Rinella M., Harrison S.A., Brunt E.M., Sanyal A.J. (2018). The diagnosis and management of nonalcoholic fatty liver disease: Practice guidance from the American Association for the Study of Liver Diseases. Hepatology.

[11] Hydes T., Alam U., Cuthbertson D.J. (2021). The Impact of Macronutrient Intake on Non-alcoholic Fatty Liver Disease (NAFLD): Too Much Fat, Too Much Carbohydrate, or Just Too Many Calories?. Front. Nutr..

[12] Katsiki N., Mikhailidis D.P., Mantzoros C.S. (2016). Non-alcoholic fatty liver disease and dyslipidemia: An update. Metabolism.

[13] Filipovic B., Marjanovic-Haljilji M., Mijac D., Lukic S., Kapor S., Kapor S., Starcevic A., Popovic D., Djokovic A. (2023). Molecular Aspects of MAFLD-New Insights on Pathogenesis and Treatment. Curr. Issues Mol. Biol..

[14] Zheng Y., Wang S., Wu J., Wang Y. (2023). Mitochondrial metabolic dysfunction and non-alcoholic fatty liver disease: New insights from pathogenic mechanisms to clinically targeted therapy. J. Transl. Med..

[15] Chen C., Zhang W., Yan G., Tang C. (2024). Identifying metabolic dysfunction-associated steatotic liver disease in patients with hypertension and pre-hypertension: An interpretable machine learning approach. Digit. Health.

[16] Younossi Z.M., Kalligeros M., Henry L. (2024). Epidemiology of Metabolic Dysfunction-Associated Steatotic Liver Disease. Clin. Mol. Hepatol..

[17] Kim D., Danpanichkul P., Wijarnpreecha K., Cholankeril G., Loomba R., Ahmed A. (2024). Current Burden of Steatotic Liver Disease and Fibrosis among Adults in the United States, 2017-2023. Clin Mol Hepatol.

[18] Gish R., Fan J.G., Dossaji Z., Fichez J., Laeeq T., Chun M., Boursier J. (2024). Review of current and new drugs for the treatment of metabolic-associated fatty liver disease. Hepatol. Int..

[19] Chen T., Qin X., Jiang J., He B. (2024). Diagnostic indicators and lifestyle interventions of metabolic-associated fatty liver disease. Front. Nutr..

[20] Chan W.K., Chuah K.H., Rajaram R.B., Lim L.L., Ratnasingam J., Vethakkan S.R. (2023). Metabolic Dysfunction-Associated Steatotic Liver Disease (MASLD): A State-of-the-Art Review. J. Obes. Metab. Syndr..

[21] Sanyal A.J., Chalasani N., Kowdley K.V., McCullough A., Diehl A.M., Bass N.M., Neuschwander-Tetri B.A., Lavine J.E., Tonascia J., Unalp A. (2010). Pioglitazone, vitamin E, or placebo for nonalcoholic steatohepatitis. N. Engl. J. Med..

[22] Parry S.A., Turner M.C., Hodson L. (2020). Lifestyle interventions affecting hepatic fatty acid metabolism. Curr. Opin. Clin. Nutr. Metab. Care.

[23] Akhavan Rezayat A., Dadgar Moghadam M., Ghasemi Nour M., Shirazinia M., Ghodsi H., Rouhbakhsh Zahmatkesh M.R., Tavakolizadeh Noghabi M., Hoseini B., Akhavan Rezayat K. (2018). Association between smoking and non-alcoholic fatty liver disease: A systematic review and meta-analysis. SAGE Open Med..

[24] Charatcharoenwitthaya P., Karaketklang K., Aekplakorn W. (2020). Cigarette Smoking Increased Risk of Overall Mortality in Patients With Non-alcoholic Fatty Liver Disease: A Nationwide Population-Based Cohort Study. Front. Med..

[25] Jeong S., Oh Y.H., Ahn J.C., Choi S., Park S.J., Kim H.J., Lee G., Son J.S., Jang H., Lee D.H. (2024). Evolutionary changes in metabolic dysfunction-associated steatotic liver disease and risk of hepatocellular carcinoma: A nationwide cohort study. Clin. Mol. Hepatol..

[26] Martins-Green M., Adhami N., Frankos M., Valdez M., Goodwin B., Lyubovitsky J., Dhall S., Garcia M., Egiebor I., Martinez B. (2014). Cigarette smoke toxins deposited on surfaces: Implications for human health. PLoS ONE.

[27] Yang D., Kim J.W., Jeong H., Kim M.S., Lim C.W., Lee K., Kim B. (2023). Effects of maternal cigarette smoke exposure on the progression of nonalcoholic steatohepatitis in offspring mice. Toxicol. Res..

[28] Sakugawa H., Nakayoshi T., Kobashigawa K., Yamashiro T., Maeshiro T., Miyagi S., Shiroma J., Toyama A., Nakayoshi T., Kinjo F. (2005). Clinical usefulness of biochemical markers of liver fibrosis in patients with nonalcoholic fatty liver disease. World J. Gastroenterol..

[29] Schwabe R.F., Tabas I., Pajvani U.B. (2020). Mechanisms of Fibrosis Development in Nonalcoholic Steatohepatitis. Gastroenterology.

[30] Munsterman I.D., Smits M.M., Andriessen R., van Nieuwkerk C.M.J., Bloemena E., Mulder C.J.J., Tjwa E., van Geenen E.J.M. (2017). Smoking is associated with severity of liver fibrosis but not with histological severity in nonalcoholic fatty liver disease. Results from a cross-sectional study. Scand. J. Gastroenterol..

[31] Germani G., Laryea M., Rubbia-Brandt L., Egawa H., Burra P., OʼGrady J., Watt K.D. (2019). Management of Recurrent and De Novo NAFLD/NASH After Liver Transplantation. Transplantation.

[32] Tincopa M.A., Anstee Q.M., Loomba R. (2024). New and emerging treatments for metabolic dysfunction-associated steatohepatitis. Cell Metab..

[33] Zeng J., Fan J.G., Francque S.M. (2024). Therapeutic management of metabolic dysfunction associated steatotic liver disease. United Eur. Gastroenterol. J..

[34] Leithead J.A., Ferguson J.W., Hayes P.C. (2008). Smoking-related morbidity and mortality following liver transplantation. Liver Transpl..

[35] Takenaka H., Fujita T., Masuda A., Yano Y., Watanabe A., Kodama Y. (2020). Non-Alcoholic Fatty Liver Disease Is Strongly Associated with Smoking Status and Is Improved by Smoking Cessation in Japanese Males: A Retrospective Study. Kobe J. Med. Sci..

[36] Mumtaz H., Hameed M., Sangah A.B., Zubair A., Hasan M. (2022). Association between smoking and non-alcoholic fatty liver disease in Southeast Asia. Front. Public Health.

[37] Ipsen D.H., Lykkesfeldt J., Tveden-Nyborg P. (2018). Molecular mechanisms of hepatic lipid accumulation in non-alcoholic fatty liver disease. Cell Mol. Life Sci..

[38] Han M., Jeong S., Song J., Park S.J., Min Lee C., Lee K., Park S.M. (2023). Association between the dual use of electronic and conventional cigarettes and NAFLD status in Korean men. Tob. Induc. Dis..

[39] Hellerstein M.K., Benowitz N.L., Neese R.A., Schwartz J.M., Hoh R., Jacob P., Hsieh J., Faix D. (1994). Effects of cigarette smoking and its cessation on lipid metabolism and energy expenditure in heavy smokers. J. Clin. Investig..

[40] Pettinelli P., Fernández T., Aguirre C., Barrera F., Riquelme A., Fernández-Verdejo R. (2023). Prevalence of non-alcoholic fatty liver disease and its association with lifestyle habits in adults in Chile: A cross-sectional study from the National Health Survey 2016-2017. Br. J. Nutr..

[41] Wang J., Li H., Wang X., Shi R., Hu J., Zeng X., Luo H., Yang P., Luo H., Cao Y. (2024). Association between triglyceride to high-density lipoprotein cholesterol ratio and nonalcoholic fatty liver disease and liver fibrosis in American adults: An observational study from the National Health and Nutrition Examination Survey 2017-2020. Front. Endocrinol..

[42] Liu J., Guan L., Zhao M., Li Q., Song A., Gao L., Lin H., Zhao J. (2021). Association Between the Triglyceride-Glucose Index and Outcomes of Nonalcoholic Fatty Liver Disease: A Large-Scale Health Management Cohort Study. Diabetes Metab. Syndr. Obes..

[43] Kurumbail R.G., Calabrese M.F. (2016). Structure and Regulation of AMPK. Exp. Suppl..

[44] Marcondes-de-Castro I.A., Reis-Barbosa P.H., Marinho T.S., Aguila M.B., Mandarim-de-Lacerda C.A. (2023). AMPK/mTOR pathway significance in healthy liver and non-alcoholic fatty liver disease and its progression. J. Gastroenterol. Hepatol..

[45] Meng Z., Liu Q., Sun F., Qiao L. (2019). Hepatitis C virus nonstructural protein 5A perturbs lipid metabolism by modulating AMPK/SREBP-1c signaling. Lipids Health Dis..

[46] Garcia D., Shaw R.J. (2017). AMPK: Mechanisms of Cellular Energy Sensing and Restoration of Metabolic Balance. Mol. Cell.

[47] Yuan H., Shyy J.Y., Martins-Green M. (2009). Second-hand smoke stimulates lipid accumulation in the liver by modulating AMPK and SREBP-1. J. Hepatol..

[48] Shimano H., Horton J.D., Shimomura I., Hammer R.E., Brown M.S., Goldstein J.L. (1997). Isoform 1c of sterol regulatory element binding protein is less active than isoform 1a in livers of transgenic mice and in cultured cells. J. Clin. Investig..

[49] Li Y., Xu S., Mihaylova M.M., Zheng B., Hou X., Jiang B., Park O., Luo Z., Lefai E., Shyy J.Y. (2011). AMPK phosphorylates and inhibits SREBP activity to attenuate hepatic steatosis and atherosclerosis in diet-induced insulin-resistant mice. Cell Metab..

[50] Horton J.D., Goldstein J.L., Brown M.S. (2002). SREBPs: Activators of the complete program of cholesterol and fatty acid synthesis in the liver. J. Clin. Investig..

[51] Osborne T.F. (2000). Sterol regulatory element-binding proteins (SREBPs): Key regulators of nutritional homeostasis and insulin action. J. Biol. Chem..

[52] Yang F.C., Xu F., Wang T.N., Chen G.X. (2021). Roles of vitamin A in the regulation of fatty acid synthesis. World J. Clin. Cases.

[53] Liu X.L., Cao H.X., Wang B.C., Xin F.Z., Zhang R.N., Zhou D., Yang R.X., Zhao Z.H., Pan Q., Fan J.G. (2017). miR-192-5p regulates lipid synthesis in non-alcoholic fatty liver disease through SCD-1. World J. Gastroenterol..

[54] Davies S.P., Sim A.T., Hardie D.G. (1990). Location and function of three sites phosphorylated on rat acetyl-CoA carboxylase by the AMP-activated protein kinase. Eur. J. Biochem..

[55] Winder W.W., Wilson H.A., Hardie D.G., Rasmussen B.B., Hutber C.A., Call G.B., Clayton R.D., Conley L.M., Yoon S., Zhou B. (1997). Phosphorylation of rat muscle acetyl-CoA carboxylase by AMP-activated protein kinase and protein kinase A. J. Appl. Physiol. (1985).

[56] Wang Q., Liu S., Zhai A., Zhang B., Tian G. (2018). AMPK-Mediated Regulation of Lipid Metabolism by Phosphorylation. Biol. Pharm. Bull..

[57] Horn C.L., Morales A.L., Savard C., Farrell G.C., Ioannou G.N. (2022). Role of Cholesterol-Associated Steatohepatitis in the Development of NASH. Hepatol. Commun..

[58] Schick S., Glantz S. (2005). Philip Morris toxicological experiments with fresh sidestream smoke: More toxic than mainstream smoke. Tob. Control.

[59] Diethelm P.A., Rielle J.C., McKee M. (2005). The whole truth and nothing but the truth? The research that Philip Morris did not want you to see. Lancet.

[60] Hasan K.M., Friedman T.C., Shao X., Parveen M., Sims C., Lee D.L., Espinoza-Derout J., Sinha-Hikim I., Sinha-Hikim A.P. (2019). E-cigarettes and Western Diet: Important Metabolic Risk Factors for Hepatic Diseases. Hepatology.

[61] Imai S., Armstrong C.M., Kaeberlein M., Guarente L. (2000). Transcriptional silencing and longevity protein Sir2 is an NAD-dependent histone deacetylase. Nature.

[62] Shen Z., Liang X., Rogers C.Q., Rideout D., You M. (2010). Involvement of adiponectin-SIRT1-AMPK signaling in the protective action of rosiglitazone against alcoholic fatty liver in mice. Am. J. Physiol. Gastrointest. Liver Physiol..

[63] Cantó C., Gerhart-Hines Z., Feige J.N., Lagouge M., Noriega L., Milne J.C., Elliott P.J., Puigserver P., Auwerx J. (2009). AMPK regulates energy expenditure by modulating NAD+ metabolism and SIRT1 activity. Nature.

[64] Nagappan A., Kim J.H., Jung D.Y., Jung M.H. (2019). Cryptotanshinone from the Salvia miltiorrhiza Bunge Attenuates Ethanol-Induced Liver Injury by Activation of AMPK/SIRT1 and Nrf2 Signaling Pathways. Int. J. Mol. Sci..

[65] Anggreini P., Kuncoro H., Sumiwi S.A., Levita J. (2023). Role of the AMPK/SIRT1 pathway in non-alcoholic fatty liver disease (Review). Mol. Med. Rep..

[66] Ponugoti B., Kim D.H., Xiao Z., Smith Z., Miao J., Zang M., Wu S.Y., Chiang C.M., Veenstra T.D., Kemper J.K. (2010). SIRT1 deacetylates and inhibits SREBP-1C activity in regulation of hepatic lipid metabolism. J. Biol. Chem..

[67] Rodgers J.T., Lerin C., Haas W., Gygi S.P., Spiegelman B.M., Puigserver P. (2005). Nutrient control of glucose homeostasis through a complex of PGC-1alpha and SIRT1. Nature.

[68] Lagouge M., Argmann C., Gerhart-Hines Z., Meziane H., Lerin C., Daussin F., Messadeq N., Milne J., Lambert P., Elliott P. (2006). Resveratrol improves mitochondrial function and protects against metabolic disease by activating SIRT1 and PGC-1alpha. Cell.

[69] Chen X., Ji Y., Liu R., Zhu X., Wang K., Yang X., Liu B., Gao Z., Huang Y., Shen Y. (2023). Mitochondrial dysfunction: Roles in skeletal muscle atrophy. J. Transl. Med..

[70] Meng D., Zhang F., Yu W., Zhang X., Yin G., Liang P., Feng Y., Chen S., Liu H. (2023). Biological Role and Related Natural Products of SIRT1 in Nonalcoholic Fatty Liver. Diabetes Metab. Syndr. Obes..

[71] Hasan M.K., Friedman T.C., Sims C., Lee D.L., Espinoza-Derout J., Ume A., Chalfant V., Lee M.L., Sinha-Hikim I., Lutfy K. (2018). α7-Nicotinic Acetylcholine Receptor Agonist Ameliorates Nicotine Plus High-Fat Diet-Induced Hepatic Steatosis in Male Mice by Inhibiting Oxidative Stress and Stimulating AMPK Signaling. Endocrinology.

[72] Han X., Bao X., Lou Q., Xie X., Zhang M., Zhou S., Guo H., Jiang G., Shi Q. (2019). Nicotinamide riboside exerts protective effect against aging-induced NAFLD-like hepatic dysfunction in mice. PeerJ.

[73] Rivera J.C., Espinoza-Derout J., Hasan K.M., Molina-Mancio J., Martínez J., Lao C.J., Lee M.L., Lee D.L., Wilson J., Sinha-Hikim A.P. (2024). Hepatic steatosis induced by nicotine plus Coca-Cola™ is prevented by nicotinamide riboside (NR). Front. Endocrinol..

[74] Willemin M.E., Lumen A. (2017). Thiocyanate: A review and evaluation of the kinetics and the modes of action for thyroid hormone perturbations. Crit. Rev. Toxicol..

[75] Aydin H.H., Celik H.A., Ersoz B. (2002). Role of thiocyanate ion in metallothionein induction and in endogenous distribution of essential elements in the rat liver. Biol. Trace Elem. Res..

[76] Vähäkangas K., Pelkonen O., Sotaniemi E. (1983). Cigarette smoking and drug metabolism. Clin. Pharmacol. Ther..

[77] Premkumar M., Anand A.C. (2021). Tobacco, Cigarettes, and the Liver: The Smoking Gun. J. Clin. Exp. Hepatol..

[78] Wang Z., Nicholls S.J., Rodriguez E.R., Kummu O., Hörkkö S., Barnard J., Reynolds W.F., Topol E.J., DiDonato J.A., Hazen S.L. (2007). Protein carbamylation links inflammation, smoking, uremia and atherogenesis. Nat. Med..

[79] Bathish B., Robertson H., Dillon J.F., Dinkova-Kostova A.T., Hayes J.D. (2022). Nonalcoholic steatohepatitis and mechanisms by which it is ameliorated by activation of the CNC-bZIP transcription factor Nrf2. Free Radic. Biol. Med..

[80] Ulasov A.V., Rosenkranz A.A., Georgiev G.P., Sobolev A.S. (2022). Nrf2/Keap1/ARE signaling: Towards specific regulation. Life Sci..

[81] Hu L., Tian K., Zhang T., Fan C.H., Zhou P., Zeng D., Zhao S., Li L.S., Smith H.S., Li J. (2019). Cyanate Induces Oxidative Stress Injury and Abnormal Lipid Metabolism in Liver through Nrf2/HO-1. Molecules.

[82] Wu Y., Song P., Zhang W., Liu J., Dai X., Liu Z., Lu Q., Ouyang C., Xie Z., Zhao Z. (2015). Activation of AMPKα2 in adipocytes is essential for nicotine-induced insulin resistance in vivo. Nat. Med..

[83] Lindell G., Farnebo L.O., Chen D., Nexø E., Rask Madsen J., Bukhave K., Graffner H. (1993). Acute effects of smoking during modified sham feeding in duodenal ulcer patients. An analysis of nicotine, acid secretion, gastrin, catecholamines, epidermal growth factor, prostaglandin E2, and bile acids. Scand. J. Gastroenterol..

[84] Li Y., Nicholson R.J., Summers S.A. (2022). Ceramide signaling in the gut. Mol. Cell Endocrinol..

[85] Park W.J., Song J.H., Kim G.T., Park T.S. (2020). Ceramide and Sphingosine 1-Phosphate in Liver Diseases. Mol. Cells.

[86] Chen B., Sun L., Zeng G., Shen Z., Wang K., Yin L., Xu F., Wang P., Ding Y., Nie Q. (2022). Gut bacteria alleviate smoking-related NASH by degrading gut nicotine. Nature.

[87] Dangana E.O., Michael O.S., Omolekulo T.E., Areola E.D., Olatunji L.A. (2019). Enhanced hepatic glycogen synthesis and suppressed adenosine deaminase activity by lithium attenuates hepatic triglyceride accumulation in nicotine-exposed rats. Biomed. Pharmacother..

[88] Qin D., Pan P., Lyu B., Chen W., Gao Y. (2024). Lupeol improves bile acid metabolism and metabolic dysfunction-associated steatotic liver disease in mice via FXR signaling pathway and gut-liver axis. Biomed. Pharmacother..

[89] Mikhail M.M., M E.L.-S., Sidrak M.S., Ghoneim M.T. (1979). Effect of chronic exposure to tobacco smoke on some blood lipids and coagulation parameters in the rat. Pharmazie.

[90] Moffatt R.J., Chelland S.A., Pecott D.L., Stamford B.A. (2004). Acute exposure to environmental tobacco smoke reduces HDL-C and HDL2-C. Prev. Med..

[91] Zvintzou E., Xepapadaki E., Skroubis G., Mparnia V., Giannatou K., Benabdellah K., Kypreos K.E. (2023). High-Density Lipoprotein in Metabolic Disorders and Beyond: An Exciting New World Full of Challenges and Opportunities. Pharmaceuticals.

[92] Souza L.L., Rossetti C.L., Peixoto T.C., Manhães A.C., de Moura E.G., Lisboa P.C. (2023). Neonatal nicotine exposure affects adult rat hepatic pathways involved in endoplasmic reticulum stress and macroautophagy in a sex-dependent manner. J. Dev. Orig. Health Dis..

[93] Li G., Chan Y.L., Wang B., Saad S., Oliver B.G., Chen H. (2020). Replacing smoking with vaping during pregnancy: Impacts on metabolic health in mice. Reprod. Toxicol..

[94] Ter Horst K.W., Serlie M.J. (2017). Fructose Consumption, Lipogenesis, and Non-Alcoholic Fatty Liver Disease. Nutrients.

[95] Yang Q., Cui Y., Luo F., Liu X., Wang Q., Bai J., Dong F., Sun Q., Lu L., Xu H. (2018). MicroRNA-191, acting via the IRS-1/Akt signaling pathway, is involved in the hepatic insulin resistance induced by cigarette smoke extract. Environ. Sci. Pollut. Res. Int..

[96] de la Monte S.M., Tong M., Agarwal A.R., Cadenas E. (2016). Tobacco Smoke-Induced Hepatic Injury with Steatosis, Inflammation, and Impairments in Insulin and Insulin-Like Growth Factor Signaling. J. Clin. Exp. Pathol..

[97] Tarantino G., Citro V., Balsano C., Capone D. (2020). Could SCGF-Beta Levels Be Associated with Inflammation Markers and Insulin Resistance in Male Patients Suffering from Obesity-Related NAFLD?. Diagnostics.

[98] Conceição E.P., Peixoto-Silva N., Pinheiro C.R., Oliveira E., Moura E.G., Lisboa P.C. (2015). Maternal nicotine exposure leads to higher liver oxidative stress and steatosis in adult rat offspring. Food Chem. Toxicol..

[99] Novaes Soares P., Silva Tavares Rodrigues V., Cherem Peixoto T., Calvino C., Aparecida Miranda R., Pereira Lopes B., Peixoto-Silva N., Lopes Costa L., Claudio-Neto S., Christian Manhães A. (2018). Cigarette Smoke During Breastfeeding in Rats Changes Glucocorticoid and Vitamin D Status in Obese Adult Offspring. Int. J. Mol. Sci..

[100] Lu N., Mei X., Li X., Tang X., Yang G., Xiang W. (2024). Preventive effects of caffeine on nicotine plus high-fat diet-induced hepatic steatosis and gain weight: A possible explanation for why obese smokers with high coffee consumption tend to be leaner. Br. J. Nutr..

[101] Liu Y.C., Wei G., Liao Z.Q., Wang F.X., Zong C., Qiu J., Le Y., Yu Z.L., Yang S.Y., Wang H.S. (2023). Design and Synthesis of Novel Indole Ethylamine Derivatives as a Lipid Metabolism Regulator Targeting PPARα/CPT1 in AML12 Cells. Molecules.

[102] Puchalska P., Crawford P.A. (2017). Multi-dimensional Roles of Ketone Bodies in Fuel Metabolism, Signaling, and Therapeutics. Cell Metab..

[103] Miranda R.A., de Moura E.G., Soares P.N., Peixoto T.C., Lopes B.P., de Andrade C.B.V., de Oliveira E., Manhães A.C., de Faria C.C., Fortunato R.S. (2020). Thyroid redox imbalance in adult Wistar rats that were exposed to nicotine during breastfeeding. Sci. Rep..

[104] Rossetti C.L., de Oliveira Costa H.M., Barthem C.S., da Silva M.H., de Carvalho D.P., da-Silva W.S. (2019). Sexual dimorphism of liver endoplasmic reticulum stress susceptibility in prepubertal rats and the effect of sex steroid supplementation. Exp. Physiol..

[105] Zhang Y., Cao C., Du S., Fan L., Zhang D., Wang X., He M. (2021). Estrogen Regulates Endoplasmic Reticulum Stress-Mediated Apoptosis by ERK-p65 Pathway to Promote Endometrial Angiogenesis. Reprod. Sci..

[106] Quraishi R., Jain R., Balhara Y.P. (2014). Profile of nicotine use among alcohol dependent patients visiting a tertiary care center in north India. Indian. J. Psychol. Med..

[107] Bailey S.M., Mantena S.K., Millender-Swain T., Cakir Y., Jhala N.C., Chhieng D., Pinkerton K.E., Ballinger S.W. (2009). Ethanol and tobacco smoke increase hepatic steatosis and hypoxia in the hypercholesterolemic apoE(-/-) mouse: Implications for a “multihit” hypothesis of fatty liver disease. Free Radic. Biol. Med..

[108] Chen X., Wang K., Cederbaum A.I., Lu Y. (2019). Suppressed hepatocyte proliferation via a ROS-HNE-P21 pathway is associated with nicotine- and cotinine-enhanced alcoholic fatty liver in mice. Biochem. Biophys. Res. Commun..

[109] Marek G.W., Malhi H. (2024). MetALD: Does it require a different therapeutic option?. Hepatology.

[110] Adner N., Nygren A. (1986). Insulin secretion in alcoholics in a withdrawal state. Acta Med. Scand..

[111] Iturriaga H., Kelly M., Bunout D., Pino M.E., Pereda T., Barrera R., Petermann M., Ugarte G. (1986). Glucose tolerance and the insulin response in recently drinking alcoholic patients: Possible effects of withdrawal. Metabolism.

[112] An H., Jang Y., Choi J., Hur J., Kim S., Kwon Y. (2025). New Insights into AMPK, as a Potential Therapeutic Target in Metabolic Dysfunction-Associated Steatotic Liver Disease and Hepatic Fibrosis. Biomol. Ther..

[113] Purohit V., Rapaka R., Kwon O.S., Song B.J. (2013). Roles of alcohol and tobacco exposure in the development of hepatocellular carcinoma. Life Sci..

[114] Ashakumary L., Vijayammal P.L. (1996). Additive effect of alcohol and nicotine on lipid peroxidation and antioxidant defence mechanism in rats. J. Appl. Toxicol..

[115] Wiśniewska E., Dylik A., Kulza M., Florek E., Piekoszewski W., Seńczuk-Przybyłowska M., Marszałek A. (2013). Exposure to ethanol and tobacco smoke in relation to level of PCNA antigen expression in pancreatic and hepatic rat cells. Pharmacol. Rep..

[116] Fouda S., Khan A., Chan S.M.H., Mahzari A., Zhou X., Qin C.X., Vlahos R., Ye J.-M. (2021). Exposure to cigarette smoke precipitates simple hepatosteatosis to NASH in high-fat diet fed mice by inducing oxidative stress. Clin. Sci..

[117] Park S., Kim J.W., Yun H., Choi S.-J., Lee S.-H., Choi K.-C., Lim C.W., Lee K., Kim B. (2016). Mainstream cigarette smoke accelerates the progression of nonalcoholic steatohepatitis by modulating Kupffer cell-mediated hepatocellular apoptosis in adolescent mice. Toxicol. Lett..

[118] Oni E.T., Figueredo V., Aneni E., Veladar E., McEvoy J.W., Blaha M.J., Blumenthal R.S., Conceicao R.D., Carvalho J.A.M., Santos R.D. (2020). Non-Alcoholic Fatty Liver Disease Modifies Serum Gamma-Glutamyl Transferase in Cigarette Smokers. J. Clin. Med. Res..

[119] Reis R., Kolci K., Bahcivan İ., Coskun G.P., Sipahi H. (2023). Alpha-Lipoic Acid Modulates the Oxidative and Inflammatory Responses Induced by Traditional and Novel Tobacco Products in Human Liver Epithelial Cells. Chem. Biodivers..

[120] Li X., Yuan L., Wang F. (2024). Health outcomes of electronic cigarettes. Chin. Med. J..

[121] Tian Y., Wang H., Han S., Fu Y., Lu F., Wang W., Li X., Ma S., Feng P., Shi Z. (2024). Liver toxicity in rats after subchronic exposure to HTP aerosol and cigarette smoke. Toxicol. Res..

[122] Adhami N., Chen Y., Martins-Green M. (2017). Biomarkers of disease can be detected in mice as early as 4 weeks after initiation of exposure to third-hand smoke levels equivalent to those found in homes of smokers. Clin. Sci..

[123] Liu J., Liang Q., Frost-Pineda K., Muhammad-Kah R., Rimmer L., Roethig H., Mendes P., Sarkar M. (2011). Relationship between biomarkers of cigarette smoke exposure and biomarkers of inflammation, oxidative stress, and platelet activation in adult cigarette smokers. Cancer Epidemiol. Biomark. Prev..

[124] Kim J.W., Zhou Z., Yun H., Park S., Choi S.J., Lee S.H., Lim C.W., Lee K., Kim B. (2020). Cigarette smoking differentially regulates inflammatory responses in a mouse model of nonalcoholic steatohepatitis depending on exposure time point. Food Chem. Toxicol..

[125] Azzalini L., Ferrer E., Ramalho L.N., Moreno M., Domínguez M., Colmenero J., Peinado V.I., Barberà J.A., Arroyo V., Ginès P. (2010). Cigarette Smoking Exacerbates Nonalcoholic Fatty Liver Disease in Obese Rats. Hepatology.

[126] Shen G.X. (2010). Oxidative stress and diabetic cardiovascular disorders: Roles of mitochondria and NADPH oxidase. Can. J. Physiol. Pharmacol..

[127] Wei Y., Pan T., Zhao Y., Chen Z., Wu L., Fang S., Wang X., Wang X., Chen D., Chen Y. (2024). Nicotine aggravates high-fat diet-induced non-alcoholic fatty liver disease in mice via inhibition of CISD3. Int. Immunopharmacol..

[128] Kanithi M., Junapudi S., Shah S.I., Matta Reddy A., Ullah G., Chidipi B. (2022). Alterations of Mitochondrial Network by Cigarette Smoking and E-Cigarette Vaping. Cells.

[129] Espinoza-Derout J., Shao X.M., Bankole E., Hasan K.M., Mtume N., Liu Y., Sinha-Hikim A.P., Friedman T.C. (2019). Hepatic DNA Damage Induced by Electronic Cigarette Exposure Is Associated With the Modulation of NAD+/PARP1/SIRT1 Axis. Front. Endocrinol..

[130] Liu W., Baker R.D., Bhatia T., Zhu L., Baker S.S. (2016). Pathogenesis of nonalcoholic steatohepatitis. Cell. Mol. Life Sci..

[131] Zhou Z., Kim J.W., Zhao J., Qi J., Choi S.J., Lim C.W., Lee M.-Y., Lee K., Kim B. (2018). Treatment of cigarette smoke extract and condensate differentially potentiates palmitic acid-induced lipotoxicity and steatohepatitis in vitro. Toxicol. Vitr..

[132] Savari F., Mard S.A., Badavi M., Rezaie A., Gharib-Naseri M.K. (2019). A new method to induce nonalcoholic steatohepatitis (NASH) in mice. BMC Gastroenterol..

[133] Martinez J.E., Kahana D.D., Ghuman S., Wilson H.P., Wilson J., Kim S.C.J., Lagishetty V., Jacobs J.P., Sinha-Hikim A.P., Friedman T.C. (2021). Unhealthy Lifestyle and Gut Dysbiosis: A Better Understanding of the Effects of Poor Diet and Nicotine on the Intestinal Microbiome. Front. Endocrinol..

[134] Magee N., Zou A., Ghosh P., Ahamed F., Delker D., Zhang Y. (2020). Disruption of hepatic small heterodimer partner induces dissociation of steatosis and inflammation in experimental nonalcoholic steatohepatitis. J. Biol. Chem..

[135] Frost-Pineda K., Liang Q., Liu J., Rimmer L., Jin Y., Feng S., Kapur S., Mendes P., Roethig H., Sarkar M. (2011). Biomarkers of potential harm among adult smokers and nonsmokers in the total exposure study. Nicotine Tob. Res..

[136] Kim J.W., Yun H., Choi S.J., Lee S.H., Park S., Lim C.W., Lee K., Kim B. (2017). Evaluating the Influence of Side Stream Cigarette Smoke at an Early Stage of Non-Alcoholic Steatohepatitis Progression in Mice. Toxicol. Res..

[137] Luo W., Xu Q., Wang Q., Wu H., Hua J. (2017). Effect of modulation of PPAR-γ activity on Kupffer cells M1/M2 polarization in the development of non-alcoholic fatty liver disease. Sci. Rep..

[138] Geng Y., Faber K.N., de Meijer V.E., Blokzijl H., Moshage H. (2021). How does hepatic lipid accumulation lead to lipotoxicity in non-alcoholic fatty liver disease?. Hepatol. Int..

[139] Kumar S., Duan Q., Wu R., Harris E.N., Su Q. (2021). Pathophysiological communication between hepatocytes and non-parenchymal cells in liver injury from NAFLD to liver fibrosis. Adv. Drug Deliv. Rev..

[140] Chow M.D., Lee Y.H., Guo G.L. (2017). The role of bile acids in nonalcoholic fatty liver disease and nonalcoholic steatohepatitis. Mol. Asp. Med..

[141] Huang S.J., Chen S.q., Lin Y., Yang H.Y., Ran J., Yan F.F., Huang M., Liu X.L., Hong L.C., Zhang X.D. (2021). Maternal nicotine exposure aggravates metabolic associated fatty liver disease via PI3K/Akt signaling in adult offspring mice. Liver Int..

[142] Hu H., Lan K., Liu H. (2023). Human symbiont Bacteroides xylanisolvens attenuates NASH through intestinal nicotine catabolism. Chin. J. Nat. Med..

[143] Meng L., Xu M., Xing Y., Chen C., Jiang J., Xu X. (2022). Effects of Cigarette Smoke Exposure on the Gut Microbiota and Liver Transcriptome in Mice Reveal Gut–Liver Interactions. Int. J. Mol. Sci..

[144] Kanda T., Goto T., Hirotsu Y., Masuzaki R., Moriyama M., Omata M. (2020). Molecular Mechanisms: Connections between Nonalcoholic Fatty Liver Disease, Steatohepatitis and Hepatocellular Carcinoma. Int. J. Mol. Sci..

[145] Kanda T., Matsuoka S., Yamazaki M., Shibata T., Nirei K., Takahashi H., Kaneko T., Fujisawa M., Higuchi T., Nakamura H. (2018). Apoptosis and non-alcoholic fatty liver diseases. World J. Gastroenterol..

[146] Guo X., Yin X., Liu Z., Wang J. (2022). Non-Alcoholic Fatty Liver Disease (NAFLD) Pathogenesis and Natural Products for Prevention and Treatment. Int. J. Mol. Sci..

[147] Xu X., Poulsen K.L., Wu L., Liu S., Miyata T., Song Q., Wei Q., Zhao C., Lin C., Yang J. (2022). Targeted therapeutics and novel signaling pathways in non-alcohol-associated fatty liver/steatohepatitis (NAFL/NASH). Signal Transduct. Target. Ther..

[148] Cawthorn W.P., Sethi J.K. (2008). TNF-alpha and adipocyte biology. FEBS Lett..

[149] Machado M.V., Michelotti G.A., Pereira Tde A., Boursier J., Kruger L., Swiderska-Syn M., Karaca G., Xie G., Guy C.D., Bohinc B. (2015). Reduced lipoapoptosis, hedgehog pathway activation and fibrosis in caspase-2 deficient mice with non-alcoholic steatohepatitis. Gut.

[150] Su Y.Q., Lin Y., Huang S.J., Lin Y.T., Ran J., Yan F.F., Liu X.L., Hong L.C., Huang M., Su H.Z. (2023). Pyroptosis is involved in maternal nicotine exposure-induced metabolic associated fatty liver disease progression in offspring mice. Mol. Reprod. Dev..

[151] Xu H.-l., Wan S.-r., An Y., Wu Q., Xing Y.-h., Deng C.-h., Zhang P.-p., Long Y., Xu B.-t., Jiang Z.-z. (2024). Targeting cell death in NAFLD: Mechanisms and targeted therapies. Cell Death Discov..

[152] Liu J., Yao Q., Xie X., Cui Q., Jiang T., Zhao Z., Du X., Lai B., Xiao L., Wang N. (2022). Procyanidin B2 Attenuates Nicotine-Induced Hepatocyte Pyroptosis through a PPARγ-Dependent Mechanism. Nutrients.

[153] Zein C.O., Unalp A., Colvin R., Liu Y.C., McCullough A.J. (2011). Smoking and severity of hepatic fibrosis in nonalcoholic fatty liver disease. J. Hepatol..

[154] Ruiz-Casas L., Pedra G., Shaikh A., Franks B., Dhillon H., Fernandes J., Mangla K.K., Augusto M., Schattenberg J.M., Romero-Gómez M. (2021). Clinical and sociodemographic determinants of disease progression in patients with nonalcoholic steatohepatitis in the United States. Medicine.

[155] She D., Jiang S., Yuan S. (2024). Association between serum cotinine and hepatic steatosis and liver fibrosis in adolescent: A population-based study in the United States. Sci. Rep..

[156] Lahelma M., Luukkonen P.K., Qadri S., Ahlholm N., Lallukka-Brück S., Porthan K., Juuti A., Sammalkorpi H., Penttilä A.K., Arola J. (2021). Assessment of Lifestyle Factors Helps to Identify Liver Fibrosis Due to Non-Alcoholic Fatty Liver Disease in Obesity. Nutrients.

[157] Zein C.O., Beatty K., Post A.B., Logan L., Debanne S., McCullough A.J. (2006). Smoking and increased severity of hepatic fibrosis in primary biliary cirrhosis: A cross validated retrospective assessment. Hepatology.

[158] Balogun O., Wang J.Y., Shaikh E.S., Liu K., Stoyanova S., Memel Z.N., Schultz H., Mun L., Bertman J., Rogen C.A. (2023). Effect of combined tobacco use and type 2 diabetes mellitus on prevalent fibrosis in patients with MASLD. Hepatol. Commun..

[159] Yilmaz Y., Yonal O., Kurt R., Avsar E. (2010). Cigarette smoking is not associated with specific histological features or severity of nonalcoholic fatty liver disease. Hepatology.

[160] Meurer S.K., Weiskirchen S., Tag C.G., Weiskirchen R. (2023). Isolation, Purification, and Culture of Primary Murine Hepatic Stellate Cells: An Update. Methods Mol. Biol..

[161] Mustra Rakic J., Liu C., Veeramachaneni S., Wu D., Paul L., Ausman L.M., Wang X.D. (2021). Dietary lycopene attenuates cigarette smoke-promoted nonalcoholic steatohepatitis by preventing suppression of antioxidant enzymes in ferrets. J. Nutr. Biochem..

[162] Jain D., Chaudhary P., Varshney N., Bin Razzak K.S., Verma D., Khan Zahra T.R., Janmeda P., Sharifi-Rad J., Daştan S.D., Mahmud S. (2021). Tobacco Smoking and Liver Cancer Risk: Potential Avenues for Carcinogenesis. J. Oncol..

[163] Chen J., Liu J., Lei Y., Liu M. (2020). Potential ameliorative effects of epigallocatechin-3-gallate against cigarette smoke exposure induced renal and hepatic deficits. Ecotoxicol. Environ. Saf..

[164] Stec D.E., Gordon D.M., Hipp J.A., Hong S., Mitchell Z.L., Franco N.R., Robison J.W., Anderson C.D., Stec D.F., Hinds T.D. (2019). Loss of hepatic PPARα promotes inflammation and serum hyperlipidemia in diet-induced obesity. Am. J. Physiol. Regul. Integr. Comp. Physiol..

[165] Kumar V., Xin X., Ma J., Tan C., Osna N., Mahato R.I. (2021). Therapeutic targets, novel drugs, and delivery systems for diabetes associated NAFLD and liver fibrosis. Adv. Drug Deliv. Rev..

[166] Hajiasgharzadeh K., Shahabi P., Karimi-Sales E., Alipour M.R. (2024). Effects of nicotine on microRNA-124 expression in bile duct ligation-induced liver fibrosis in rats. BMC Pharmacol. Toxicol..

[167] Li M., Chen L., Gao Y., Li M., Wang X., Qiang L., Wang X. (2020). Recent advances targeting C-C chemokine receptor type 2 for liver diseases in monocyte/macrophage. Liver Int..

[168] Soeda J., Morgan M., McKee C., Mouralidarane A., Lin C., Roskams T., Oben J.A. (2012). Nicotine induces fibrogenic changes in human liver via nicotinic acetylcholine receptors expressed on hepatic stellate cells. Biochem. Biophys. Res. Commun..

[169] Munker S., Wu Y.L., Ding H.G., Liebe R., Weng H.L. (2017). Can a fibrotic liver afford epithelial-mesenchymal transition?. World J. Gastroenterol..

[170] Dewidar B., Meyer C., Dooley S., Meindl-Beinker A.N. (2019). TGF-β in Hepatic Stellate Cell Activation and Liver Fibrogenesis-Updated 2019. Cells.

[171] Liang Z., Wu R., Xie W., Xie C., Wu J., Geng S., Li X., Zhu M., Zhu W., Zhu J. (2017). Effects of Curcumin on Tobacco Smoke-induced Hepatic MAPK Pathway Activation and Epithelial-Mesenchymal Transition In Vivo. Phytother. Res..

[172] Jensen K., Afroze S., Ueno Y., Rahal K., Frenzel A., Sterling M., Guerrier M., Nizamutdinov D., Dostal D.E., Meng F. (2013). Chronic nicotine exposure stimulates biliary growth and fibrosis in normal rats. Dig. Liver Dis..

[173] Siapoush S., Rezaei R., Alavifard H., Hatami B., Zali M.R., Vosough M., Lorzadeh S., Łos M.J., Baghaei K., Ghavami S. (2023). Therapeutic implications of targeting autophagy and TGF-β crosstalk for the treatment of liver fibrosis. Life Sci..

[174] Song Y., Wei J., Li R., Fu R., Han P., Wang H., Zhang G., Li S., Chen S., Liu Z. (2023). Tyrosine kinase receptor B attenuates liver fibrosis by inhibiting TGF-β/SMAD signaling. Hepatology.

[175] Fabregat I., Moreno-Càceres J., Sánchez A., Dooley S., Dewidar B., Giannelli G., Ten Dijke P. (2016). TGF-β signalling and liver disease. Febs J.

[176] Barcellos-Hoff M.H., Dix T.A. (1996). Redox-mediated activation of latent transforming growth factor-beta 1. Mol. Endocrinol..

[177] Hu H.H., Chen D.Q., Wang Y.N., Feng Y.L., Cao G., Vaziri N.D., Zhao Y.Y. (2018). New insights into TGF-β/Smad signaling in tissue fibrosis. Chem. Biol. Interact..

[178] Jiang F., Liu G.S., Dusting G.J., Chan E.C. (2014). NADPH oxidase-dependent redox signaling in TGF-β-mediated fibrotic responses. Redox Biol..

[179] Xu F., Liu C., Zhou D., Zhang L. (2016). TGF-β/SMAD Pathway and Its Regulation in Hepatic Fibrosis. J. Histochem. Cytochem..

[180] Massagué J. (2000). How cells read TGF-beta signals. Nat. Rev. Mol. Cell Biol..

[181] Higashi T., Friedman S.L., Hoshida Y. (2017). Hepatic stellate cells as key target in liver fibrosis. Adv. Drug Deliv. Rev..

[182] Khomich O., Ivanov A.V., Bartosch B. (2019). Metabolic Hallmarks of Hepatic Stellate Cells in Liver Fibrosis. Cells.

[183] Friedman S.L. (2008). Mechanisms of hepatic fibrogenesis. Gastroenterology.

[184] Yang Y., Sun M., Li W., Liu C., Jiang Z., Gu P., Li J., Wang W., You R., Ba Q. (2021). Rebalancing TGF-β/Smad7 signaling via Compound kushen injection in hepatic stellate cells protects against liver fibrosis and hepatocarcinogenesis. Clin. Transl. Med..

[185] Zhang R., Li X., Gao Y., Tao Q., Lang Z., Zhan Y., Li C., Zheng J. (2023). Ginsenoside Rg1 Epigenetically Modulates Smad7 Expression in Liver Fibrosis via MicroRNA-152. J. Ginseng Res..

[186] Yao Q.Y., Xu B.L., Wang J.Y., Liu H.C., Zhang S.C., Tu C.T. (2012). Inhibition by curcumin of multiple sites of the transforming growth factor-beta1 signalling pathway ameliorates the progression of liver fibrosis induced by carbon tetrachloride in rats. BMC Complement. Altern. Med..

[187] Medeiros A.I., Sá-Nunes A., Soares E.G., Peres C.M., Silva C.L., Faccioli L.H. (2004). Blockade of endogenous leukotrienes exacerbates pulmonary histoplasmosis. Infect. Immun..

[188] El-Sherbeeny N.A., Nader M.A., Attia G.M., Ateyya H. (2016). Agmatine protects rat liver from nicotine-induced hepatic damage via antioxidative, antiapoptotic, and antifibrotic pathways. Naunyn Schmiedebergs Arch. Pharmacol..

[189] Xu M.-Y., Hu J.-J., Shen J., Wang M.-L., Zhang Q.-Q., Qu Y., Lu L.-G. (2014). Stat3 signaling activation crosslinking of TGF-β1 in hepatic stellate cell exacerbates liver injury and fibrosis. Biochim. Et Biophys. Acta (BBA)—Mol. Basis Dis..

[190] Zhang D., Dai J., Zhang M., Xie Y., Cao Y., He G., Xu W., Wang L., Qiao Z., Qiao Z. (2021). Paternal nicotine exposure promotes hepatic fibrosis in offspring. Toxicol. Lett..

[191] Roeb E. (2018). Matrix metalloproteinases and liver fibrosis (translational aspects). Matrix Biol..

[192] Ries C. (2014). Cytokine functions of TIMP-1. Cell Mol. Life Sci..

[193] Kawabe T.T., Rea T.J., Flenniken A.M., Williams B.R., Groppi V.E., Buhl A.E. (1991). Localization of TIMP in cycling mouse hair. Development.

[194] Murphy F.R., Issa R., Zhou X., Ratnarajah S., Nagase H., Arthur M.J., Benyon C., Iredale J.P. (2002). Inhibition of apoptosis of activated hepatic stellate cells by tissue inhibitor of metalloproteinase-1 is mediated via effects on matrix metalloproteinase inhibition: Implications for reversibility of liver fibrosis. J. Biol. Chem..

[195] Zhang J., Guo H., Zhang H., Wang H., Qian G., Fan X., Hoffman A.R., Hu J.F., Ge S. (2011). Putative tumor suppressor miR-145 inhibits colon cancer cell growth by targeting oncogene Friend leukemia virus integration 1 gene. Cancer.

[196] Huang X., Wang X., Wang Y., Shen S., Chen W., Liu T., Wang P., Fan X., Liu L., Jia J. (2024). TIMP-1 Promotes Expression of MCP-1 and Macrophage Migration by Inducing Fli-1 in Experimental Liver Fibrosis. J. Clin. Transl. Hepatol..

[197] Younossi Z.M., Zelber-Sagi S., Henry L., Gerber L.H. (2023). Lifestyle interventions in nonalcoholic fatty liver disease. Nat. Rev. Gastroenterol. Hepatol..

[198] Aizawa K., Liu C., Tang S., Veeramachaneni S., Hu K.Q., Smith D.E., Wang X.D. (2016). Tobacco carcinogen induces both lung cancer and non-alcoholic steatohepatitis and hepatocellular carcinomas in ferrets which can be attenuated by lycopene supplementation. Int. J. Cancer.

[199] Salahshoor M., Mohamadian S., Kakabaraei S., Roshankhah S., Jalili C. (2016). Curcumin improves liver damage in male mice exposed to nicotine. J. Tradit. Complement. Med..

[200] Fanoudi S., Alavi M.S., Mehri S., Hosseinzadeh H. (2024). The protective effects of curcumin against cigarette smoke-induced toxicity: A comprehensive review. Phytother. Res..

[201] Al-Awaida W., Goh K.W., Al-Ameer H.J., Gushchina Y.S., Torshin V.I., Severin A.E., Al Bawareed O., Srour B., Al Farraj J., Hamad I. (2023). Assessing the Protective Role of Epigallocatechin Gallate (EGCG) against Water-Pipe Smoke-Induced Toxicity: A Comparative Study on Gene Expression and Histopathology. Molecules.

[202] Barbosa R.J., Ratti da Silva G., Cola I.M., Kuchler J.C., Coelho N., Barboza L.N., Menetrier J.V., de Souza R., Zonta F.N., Froehlich D.L. (2020). Promising therapeutic use of Baccharis trimera (less.) DC. as a natural hepatoprotective agent against hepatic lesions that are caused by multiple risk factors. J. Ethnopharmacol..

[203] Murillo A.G., Fernandez M.L. (2016). Potential of Dietary Non-Provitamin A Carotenoids in the Prevention and Treatment of Diabetic Microvascular Complications. Adv. Nutr..

[204] Erhardt A., Stahl W., Sies H., Lirussi F., Donner A., Häussinger D. (2011). Plasma levels of vitamin E and carotenoids are decreased in patients with Nonalcoholic Steatohepatitis (NASH). Eur. J. Med. Res..

[205] Peterson L.A., Stanfill S.B., Hecht S.S. (2024). An update on the formation in tobacco, toxicity and carcinogenicity of N’-nitrosonornicotine and 4-(methylnitrosamino)-1-(3-pyridyl)-1-butanone. Carcinogenesis.

[206] Wu L., Guo X., Hartson S.D., Davis M.A., He H., Medeiros D.M., Wang W., Clarke S.L., Lucas E.A., Smith B.J. (2017). Lack of β, β-carotene-9′, 10′-oxygenase 2 leads to hepatic mitochondrial dysfunction and cellular oxidative stress in mice. Mol. Nutr. Food Res..

[207] Lim J.Y., Liu C., Hu K.Q., Smith D.E., Wang X.D. (2018). Ablation of carotenoid cleavage enzymes (BCO1 and BCO2) induced hepatic steatosis by altering the farnesoid X receptor/miR-34a/sirtuin 1 pathway. Arch. Biochem. Biophys..

[208] Willems P.H., Rossignol R., Dieteren C.E., Murphy M.P., Koopman W.J. (2015). Redox Homeostasis and Mitochondrial Dynamics. Cell Metab..

[209] Qiu X., Brown K., Hirschey M.D., Verdin E., Chen D. (2010). Calorie restriction reduces oxidative stress by SIRT3-mediated SOD2 activation. Cell Metab..

[210] Agarwal A., Nallella K.P., Allamaneni S.S., Said T.M. (2004). Role of antioxidants in treatment of male infertility: An overview of the literature. Reprod. Biomed. Online.

[211] Kalpana C., Sudheer A.R., Rajasekharan K.N., Menon V.P. (2007). Comparative effects of curcumin and its synthetic analogue on tissue lipid peroxidation and antioxidant status during nicotine-induced toxicity. Singap. Med. J..

[212] Kwapisz O., Górka J., Korlatowicz A., Kotlinowski J., Waligórska A., Marona P., Pydyn N., Dobrucki J.W., Jura J., Miekus K. (2021). Fatty Acids and a High-Fat Diet Induce Epithelial-Mesenchymal Transition by Activating TGFβ and β-Catenin in Liver Cells. Int. J. Mol. Sci..

[213] Bimonte S., Albino V., Piccirillo M., Nasto A., Molino C., Palaia R., Cascella M. (2019). Epigallocatechin-3-gallate in the prevention and treatment of hepatocellular carcinoma: Experimental findings and translational perspectives. Drug Des. Devel Ther..

[214] O’Callaghan F., Muurlink O., Reid N. (2018). Effects of caffeine on sleep quality and daytime functioning. Risk Manag. Healthc. Policy.

[215] Saimaiti A., Zhou D.D., Li J., Xiong R.G., Gan R.Y., Huang S.Y., Shang A., Zhao C.N., Li H.Y., Li H.B. (2023). Dietary sources, health benefits, and risks of caffeine. Crit. Rev. Food Sci. Nutr..

[216] Shen H., Rodriguez A.C., Shiani A., Lipka S., Shahzad G., Kumar A., Mustacchia P. (2016). Association between caffeine consumption and nonalcoholic fatty liver disease: A systemic review and meta-analysis. Ther. Adv. Gastroenterol..

[217] Dranoff J.A. (2023). Coffee as chemoprotectant in fatty liver disease: Caffeine-dependent and caffeine-independent effects. Am. J. Physiol. Gastrointest. Liver Physiol..

[218] Xu G., Huang K., Zhou J. (2018). Hepatic AMP Kinase as a Potential Target for Treating Nonalcoholic Fatty Liver Disease: Evidence from Studies of Natural Products. Curr. Med. Chem..

[219] Fang C., Cai X., Hayashi S., Hao S., Sakiyama H., Wang X., Yang Q., Akira S., Nishiguchi S., Fujiwara N. (2019). Caffeine-stimulated muscle IL-6 mediates alleviation of non-alcoholic fatty liver disease. Biochim. Biophys. Acta Mol. Cell Biol. Lipids.

[220] Meamar M., Raise-Abdullahi P., Rashidy-Pour A., Raeis-Abdollahi E. (2024). Coffee and mental disorders: How caffeine affects anxiety and depression. Prog. Brain Res..

[221] Chaudhary N.S., Taylor B.V., Grandner M.A., Troxel W.M., Chakravorty S. (2021). The effects of caffeinated products on sleep and functioning in the military population: A focused review. Pharmacol. Biochem. Behav..

[222] Rubio-Gutierrez J.C., Mendez-Hernández P., Guéguen Y., Galichon P., Tamayo-Ortiz M., Haupt K., Medeiros M., Barbier O.C. (2022). Overview of Traditional and Environmental Factors Related to Bone Health. Environ. Sci. Pollut. Res. Int..

[223] Sonestedt E., Lukic M. (2024). Beverages—A scoping review for Nordic Nutrition Recommendations 2023. Food Nutr. Res..

[224] Verdi L.G., Brighente I.M.C., Pizzolatti M.G. (2005). The Baccharis genus (Asteraceae): Chemical, economic and biological aspects. Quim. Nova.

[225] Saygin M., Asci H., Cankara F.N., Bayram D., Yesilot S., Candan I.A., Alp H.H. (2016). The impact of high fructose on cardiovascular system: Role of α-lipoic acid. Hum. Exp. Toxicol..

[226] Salehi B., Berkay Yılmaz Y., Antika G., Boyunegmez Tumer T., Fawzi Mahomoodally M., Lobine D., Akram M., Riaz M., Capanoglu E., Sharopov F. (2019). Insights on the Use of α-Lipoic Acid for Therapeutic Purposes. Biomolecules.

[227] Gumral N., Aslankoc R., Senol N., Cankara F.N. (2021). Protective Effect of Alpha-Lipoic Acid against Liver Damage Induced by Cigarette Smoke: An in vivo Study. Saudi J. Med. Med. Sci..

